# LRRC71 is essential for sperm motility, fertilization, and male fertility

**DOI:** 10.1016/j.jbc.2026.113176

**Published:** 2026-05-20

**Authors:** Lu Yuan, Chuan Xu, Tingting Ge, Guanghua Li, Shiqi Meng, Wenting Lu, Yueqi Yang, Yichun Zhao, Xiao Fang, Ya Zhao, Changmin Niu, Xuhui Zeng, Fan Yang, Ying Zheng

**Affiliations:** 1 School of Basic Medical Sciences & School of Public Health, Faculty of Medicine, Yangzhou University, Yangzhou, Jiangsu, China; 2 Reproductive Medicine Center, Department of Obstetrics and Gynecology, The First Affiliated Hospital of Anhui Medical University, Hefei, Anhui, China; 3 School of Nursing, Faculty of Medicine, Yangzhou University, Yangzhou, Jiangsu, China; 4 Institute of Reproductive Medicine, School of Medicine, Nantong University, Nantong, Jiangsu, China; 5 Affiliated Hospital of Yangzhou University, Yangzhou University, Yangzhou, Jiangsu, China

**Keywords:** asthenozoospermia, energy metabolism, fertilization, LRRC71, male infertility, sperm motility

## Abstract

Although sperm supply half the genomic material necessary for generating viable offspring in sexual reproduction, the core molecular mechanisms underlying their formation remain largely unelucidated. Here, we demonstrate that leucine-rich repeat-containing 71 (LRRC71) is essential for spermatogenesis and male fertility in mice. *Lrrc71* knockout (KO) mice exhibit asthenozoospermia and disrupted mitochondrial sheaths in sperm. Both glycolysis and oxidative phosphorylation (OXPHOS) pathways were disrupted in *Lrrc71* KO sperm, resulting in reduced ATP content. Furthermore, *Lrrc71* KO sperm showed defective fertilization capacity and failed sperm migration into the oviduct. Key metabolic enzymes and fertilization-related proteins were significantly down-regulated in *Lrrc71* KO sperm, and co-immunoprecipitation validated their interactions with LRRC71. Also, a novel heterozygous variant of *LRRC71* was identified in a patient with asthenozoospermia. Despite failed *in vitro* fertilization (IVF), a successful intracytoplasmic sperm injection outcome was achieved in *Lrrc71* KO mice. These findings demonstrate the essential role of LRRC71 in regulating spermatogenesis and sperm function, which facilitates our understanding of the molecular mechanisms underlying spermatogenesis. LRRC71 represents a potential target for the diagnosis and treatment of male infertility associated with asthenozoospermia.

Male infertility affects approximately one in six adult men worldwide, with abnormalities in sperm concentration, motility, and morphology as primary contributing factors (1, 2). Asthenozoospermia, defined as a sperm progressive motility rate < 32%, accounts for approximately 20 to 40% of male infertility cases (1, 2, 3). Although genetic mutations in members of a-kinase anchoring protein (AKAP), tetratricopeptide repeat domain-containing (TTC), dynein, axonemal, heavy chain (DNAH), and cilia- and flagella-associated protein (CFAP) families have been linked to asthenozoospermia (3, 4, 5), numerous additional genes regulating sperm motility remain uncharacterized.

Reduced sperm progressive motility is the primary hallmark of asthenozoospermia. The adenosine triphosphate (ATP) production for sperm movement relies on the structural integrity of the flagellum and a tightly regulated energy metabolism system (6, 7). Abnormalities in the midpiece of sperm, including disrupted mitochondrial sheath assembly and defective mitochondrial sheath structure (8, 9), have been associated with impaired energy metabolism, reduced sperm motility and male infertility. However, the molecular mechanisms underlying sperm energy metabolism and motility are poorly understood.

The leucine-rich repeat-containing (LRRC) protein family is composed of evolutionarily conserved proteins that feature LRR domains capable of forming a horseshoe-like structure to mediate specific protein–protein interactions (10, 11, 12). Several LRRC family members are involved in flagellum assembly, motility regulation, and sperm physiological function. For example, LRRC50 deficiency causes ciliary defects and male infertility in humans (13), while mutations in *LRRC6*, a key axonemal assembly factor, lead to primary ciliary dyskinesia (14). LRRC56 aids in the maturation and trafficking of dynein arm complexes (15). In sperm, LRRC23 plays a crucial role in stabilizing the radial spoke complex in the axoneme, and its mutation results in asthenozoospermia (16). Additionally, LRRC52 serves as an auxiliary subunit of the pH-sensitive potassium channel Slo3, which regulates KSper current, sperm activation, and fertilization competence (17). These studies highlight the significance of LRRC proteins in sperm motility through structural, ionic, and signaling pathways, with dysfunctions leading to various asthenozoospermic phenotypes. LRRC71 is a member of LRRC family and is predominantly expressed in the mouse testis. A previous study has reported that *LRRC71* is a potential target gene of Wnt/β-catenin signaling pathway (18), which may play an important role in the diagnosis and treatment of glioblastoma multiforme. Moreover, it has been reported that *LRRC71* is a candidate gene related to milk traits in sheep (19). However, the function of LRRC71 in spermatogenesis and male fertility remains poorly understood.

In this study, we investigated the role of LRRC71 in mouse spermatogenesis and male fertility using an *Lrrc71* knockout (KO) mouse model. Meanwhile, *LRRC71* mutations were identified in patients with asthenozoospermia. Our results highlight the importance of LRRC proteins in germ cell function and define a specific role for LRRC71 in regulating spermatogenesis and sperm function, offering new molecular insights into the genetic underpinnings of asthenozoospermia.

## Results

### Testis-enriched expression of LRRC71 in mouse spermatogenesis

To investigate the evolutionary characteristics of LRRC71, phylogenetic analysis of LRRC71 proteins from nine mammalian species was performed. Human LRRC71 clustered with primate orthologs and formed a monophyletic clade with chimpanzee LRRC71 (bootstrap value = 100), which is consistent with known mammalian phylogeny (Fig. S1*A*). The expression pattern of *LRRC71* in humans was further analyzed. Public RNA-seq data (https://www.proteinatlas.org/) demonstrated that *LRRC71* exhibited the highest expression level in testicular tissue, with moderate expression in multiple ciliated organs, including the fallopian tube and lung (Fig. S1*B*). To clarify the cellular expression of *LRRC71* within the testis, single-cell transcriptome sequencing data (https://www.proteinatlas.org/) were analyzed. The results showed that within testicular tissues, *LRRC71* was predominantly expressed in early and late spermatids (Fig. S1*C*), indicating that *LRRC71* may exert a critical function during spermiogenesis. Protein sequence conservation of LRRC71 was compared across five mammalian species (https://www.uniprot.org/), and below-average conservation was observed in these lineages (Fig. S1*D*). Collectively, these findings suggest that the mouse represents an ideal model organism for investigating the function of LRRC71 in spermatogenesis.

**Figure 1:**
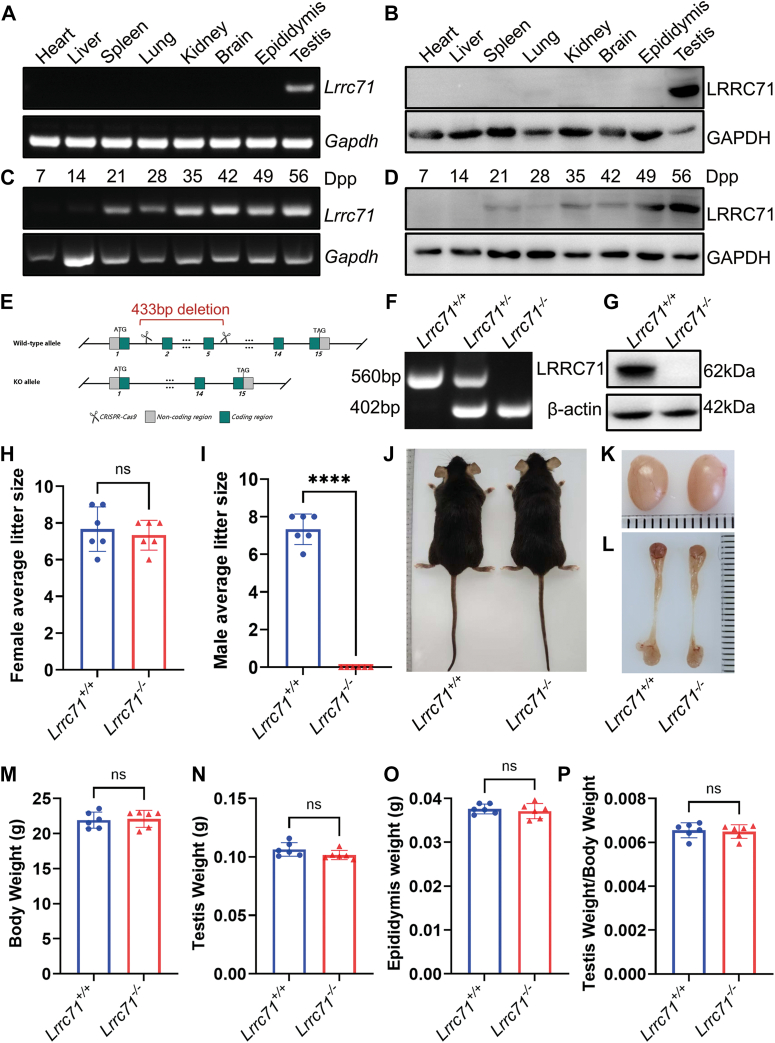
**Testis-enriched LRRC71 is essential for male fertility in mice.***A*, RT-PCR analysis of *Lrrc71* mRNA in multiple mouse tissues. *B*, Western blot analysis of LRRC71 protein in multiple mouse tissues. *C*, RT-PCR analysis of *Lrrc71* mRNA in mouse testis at different postnatal ages. *D*, Western blot analysis of LRRC71 protein in mouse testis at different postnatal ages. *E*, Schematic diagram of generating *Lrrc71*-knockout mouse model (*Lrrc71*^−/−^) using CRISPR/Cas9 system. *F*, representative PCR genotyping results of the *Lrrc71* allele in mice. Band size: wild-type (*Lrrc71*^+/+^, 560 bp), heterozygous (*Lrrc71*^+/−^, 560/402 bp), homozygous (*Lrrc71*^−/−^, 402 bp). *G*, Western blot analysis of LRRC71 protein in the testes of 8-week-old *Lrrc71*^+/+^ and *Lrrc71*^−/−^ mice. *H*, average litter size of *Lrrc71*^+/+^ male mice mated with *Lrrc71*^+/+^ and *Lrrc71*^−/−^ females (n = 6). Student’s *t* test. *I*, average litter size of *Lrrc71*^+/+^ female mice mated with *Lrrc71*^+/+^ and *Lrrc71*^−/−^ males (n = 6). Student’s *t* test. *J*, morphology of 8-week-old *Lrrc71*^+/+^ and *Lrrc71*^−/−^ mice. Morphology of (*K*) testes and (*L*) epididymis from 8-week-old mice. *M*, body weight, (*N*) testis weight, (*O*) epididymis weight, and (*P*) epididymis/body weight ratio of 8-week-old mice (*n* = 6). Student’s *t* test. Data are presented as mean ± SD. ns: no significant difference. ∗*p* < 0.05, ∗∗*p* < 0.01, ∗∗∗*p* < 0.001.

To investigate the expression pattern of *Lrrc71* in spermatogenesis of mice, reverse transcription polymerase chain reaction (RT-PCR) and Western blot analyses were performed. As shown in Figure 1, *A* and *B*, both LRRC71 mRNA and protein are predominantly expressed in the mouse testis. There was a low expression of *Lrrc71* mRNA in the testes of mice at 14 days *postpartum* (dpp), and a continuously increased *Lrrc71* expression was observed from 14 dpp to 56 dpp (Fig. 1*C*). LRRC71 protein expression was first detected in the testes of mice at 21 dpp, which coincides with the onset of round spermatid formation (Fig. 1*D*), suggesting that LRRC71 may be involved in spermiogenesis. It has been reported that *Nanos2* KO mice exhibit Sertoli cell-only syndrome, with no spermatogenic cells present in the testes (20). We evaluated LRRC71 protein expression in *Nanos2* KO mouse testes *via* Western blot and found no detectable LRRC71 expression, indicating that LRRC71 expression in the testis is likely restricted to spermatogenic cells (Fig. S1*E*). Due to the lack of a specific antibody for immunofluorescence (IF) staining, the subcellular localization of LRRC71 was investigated *in vitro*. We expressed an EGFP-LRRC71 fusion protein in four distinct cell lines: HeLa (human cervical carcinoma), NIH3T3 (mouse embryonic fibroblast), GC1-spg and GC2-spd. In all four cell lines, EGFP-LRRC71 exhibited a predominantly diffuse cytoplasmic distribution, with limited co-localization with the endoplasmic reticulum (ER), mitochondria and lysosomes (Fig. S2). Together, these data suggest that LRRC71 is evolutionarily conserved across species with testis-enriched expression.

### LRRC71 is required for male fertility in mice

To investigate the biological function of *Lrrc71*, we generated *Lrrc71* KO mice using the CRISPR-Cas9 system by deleting a 433-bp region spanning exons 2 to 5 (Fig. 1*E*). Genotyping was performed by RT-PCR (Fig. 1*F*), and the absence of LRRC71 protein in the testes of *Lrrc71* KO mice was confirmed by Western blot (Fig. 1*G*). The pairing of *Lrrc71* KO male mice with adult wild-type (WT) females for a two-month period achieved zero pups, while the WT males generated offspring when paired with adult WT females (Fig. 1*I*). Additionally, *Lrrc71* KO female mice paired with WT males generated a normal number of offspring over a two-month period (Fig. 1*H*), despite high fallopian tube expression of *LRRC71* (Fig. S1*B*). Histological analysis of oviducts revealed no significant differences between KO and WT females Fig. S3*A*), indicating normal organ structure. The size of body (Fig. 1*J*), testis (Fig. 1*K*), and epididymis (Fig. 1*L*) of *Lrrc71* KO mice was similar to that of WT mice. The body weight (Fig. 1*M*), testis weight (Fig. 1*N*), epididymis weight (Fig. 1*O*), and testis-to-body weight ratio (Fig. 1*P*) in *Lrrc71* KO mice were not changed significantly when compared with WT mice. These results demonstrate that LRRC71 is required for male fertility and suggest that deletion of *Lrrc71* does not show negative impact on other physiological systems of mice.

### Asthenoteratozoospermia in mice with genetic inactivation of *Lrrc71*

**Figure 2:**
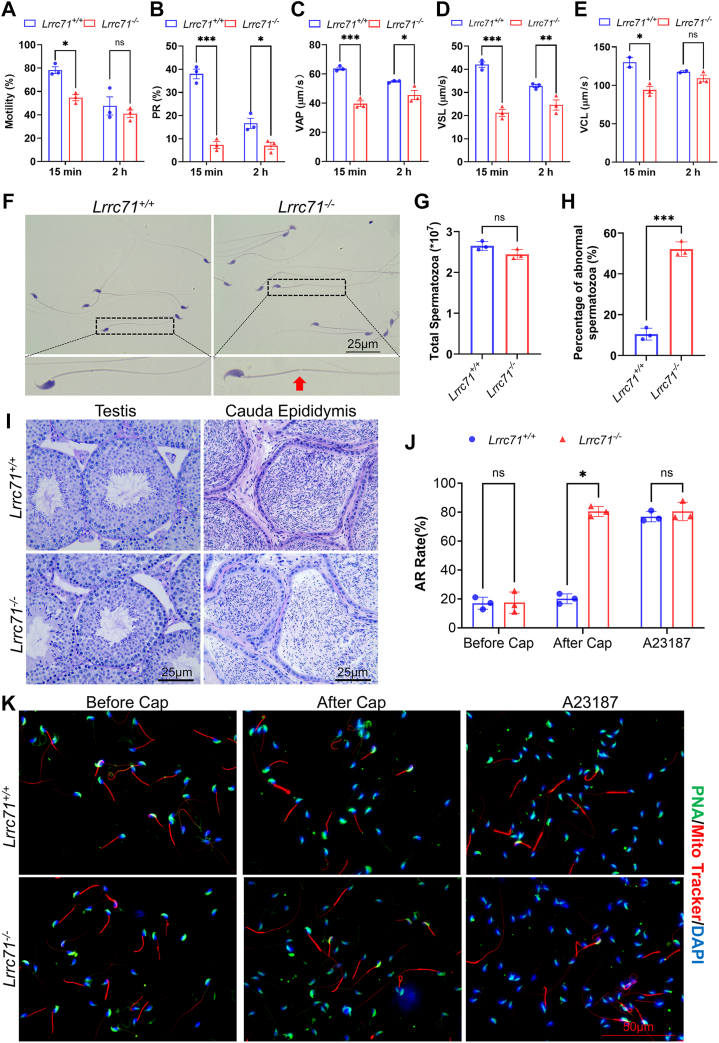
**Sperm parameter assessment of *Lrrc71*^*−/−*^ mice.** Epididymal sperm from 8-week-old mice were collected for computer-assisted sperm analysis (CASA) to detect (*A*) sperm motility, (*B*) progressive motility, (*C*) average path velocity (VAP), (*D*) straight-line velocity (VSL), and (*E*) curvilinear velocity (VCL) (n = 3). Student’s *t* test. *F*, representative images of the epididymal spermatozoa from 8-week-old mice. *G*, number of epididymal sperm in 8-week-old *Lrrc71*^*+/+*^ and *Lrrc71*^*−/−*^ mice (n = 3). Student’s *t* test. *H*, percentage of abnormal sperm in 8-week-old *Lrrc71*^*+/+*^ and *Lrrc71*^*−/−*^ mice (n = 3). Student’s *t* test. *I*, hematoxylin and eosin (H&E) staining of the testes and cauda epididymis sections from *Lrrc71*^*+/+*^ and *Lrrc71*^*−/−*^ mice. Scale bar is 25 μm. All data are presented as mean ± SD. ns: no significant difference. ∗*p* < 0.05, ∗∗*p* < 0.01, ∗∗∗*p* < 0.001. *J*, percentage of acrosome-reacted sperm (mitochondria-positive). n = 3, Student’s *t* test. *K*, epididymal sperm were collected from 8-week-old mice and incubated in HTF medium to induce capacitation (Cap), or treated with calcium ionophore A23187 to induce acrosome reaction. Then the IF staining was performed to visualize mitochondria (*red color*), acrosomes (*green color*), and nuclei (*blue color*). Scale bar is 50 μm.

To investigate the reason for male infertility in *Lrrc71* KO mice, we first analyzed the motile parameters of epididymal sperm using computer-assisted sperm analysis (CASA). Results showed that before sperm capacitation, the percentages of motile sperm (Fig. 2*A*) and progressively motile (PR) sperm (Fig. 2*B*) were significantly reduced in *Lrrc71* KO mice when compared with WT controls. Key kinematic parameters including average path velocity (VAP), straight-line velocity (VSL), and curvilinear velocity (VCL), were also significantly reduced in *Lrrc71* KO sperm (Fig. 2, *C*–*E*). After capacitation, the motility rate and VCL of KO sperm were comparable to those of WT sperm, whereas the PR rate and the motility parameters VAP and VSL were significantly lower than those of WT sperm (Fig. 2, *A*–*E*). However, the number of epididymal sperm in WT and *Lrrc71* KO mice was comparable (Fig. 2*G*). Further morphological analyses revealed that *Lrrc71* KO sperm exhibited normal morphology except for the abnormal connections between the midpiece and principal piece (Fig. 2, *F* and *H*). Histological analysis of testicular and epididymal tissues showed no obvious structural abnormalities, and all spermatogenic stages appeared morphologically normal (Figs. 2*I* and S3*B*). To further determine changes in testicular cells after *Lrrc71* deletion, proliferating cell nuclear antigen (PCNA) (21), phosphorylated histone H2AX at Ser139 (γH2AX) (22), and SRY-box transcription factor 9 (SOX9) (23) were used to mark spermatogonia, spermatocytes, and Sertoli cells, respectively. The number of spermatogonia (Fig. S4, *A* and *B*), spermatocytes (Fig. S4, *C* and *D*), and Sertoli cells (Fig. S4, *E* and *F*) in *Lrrc71* KO testes was not changed significantly when compared with WT testes, which suggests that LRRC71 deficiency does not affect spermatogonial differentiation, meiotic progression in spermatocytes, and Sertoli cell development.

To evaluate the functional role of *Lrrc71* in sperm, we further examined the acrosome reaction. Peanut agglutinin (PNA) fluorescent staining was used to assess the rate of sperm acrosome reaction. To exclude the confounding effects of dead sperm on acrosome evaluation, mitochondrial dye staining was performed simultaneously to discriminate viable and dead sperm. Only mitochondria-positive viable sperm were analyzed for the acrosome reaction rate. The results revealed no significant difference in the acrosome reaction rate between *Lrrc71* KO and WT sperm before capacitation. After capacitation, however, the acrosome reaction rate was significantly higher in KO sperm than in WT counterparts. Moreover, upon induction of the acrosome reaction with the calcium ionophore A23187 in capacitated sperm, no obvious difference in acrosome reaction rate was found between WT and KO groups, indicating that *Lrrc71*-deficient sperm undergo spontaneous acrosome reaction during capacitation (Fig. 2, *J* and *K*). In addition, we assessed the development of the acrosome and manchette during spermiogenesis in *Lrrc71* KO mice, and no morphological differences were observed relative to wild-type controls (Fig. S4*G*). These data suggest that *Lrrc71* deficiency does not impair acrosome biogenesis but instead leads to defective sperm functional activity. Collectively, our results demonstrate that LRRC71 is required for sperm morphogenesis and function, and its deficiency leads to asthenoteratozoospermia and male infertility.

### Impaired acrosome integrity and midpiece–principal piece junction in *Lrrc71* knockout sperm

**Figure 3:**
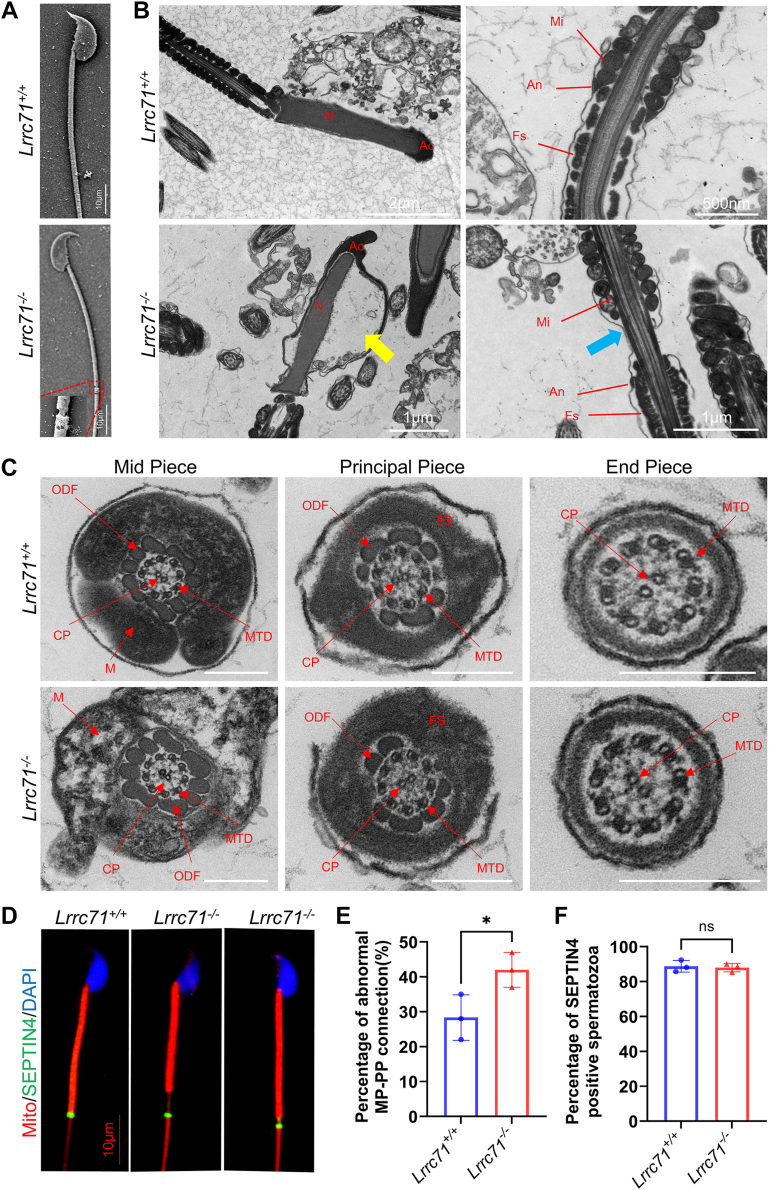
**Ultrastructure of sperm from *Lrrc71*^*−/−*^ mice.***A*, scanning electron microscopy (SEM) analysis of epididymal sperm from *Lrrc71*^*+/+*^ and *Lrrc71*^*−/−*^ mice. *B*, transmission electron microscopy (TEM) analysis of epididymal sperm from *Lrrc71*^*+/+*^ and *Lrrc71*^*−/−*^ mice. The *yellow arrow* indicates the detached acrosome from the nuclear membrane, the *blue arrow* indicates the partial absence of the mitochondrial sheath at the junction between the midpiece and principal piece of the sperm tail. Ac, acrosome; An, annulus; Fs, fiber sheath; Mi, mitochondrion; N, nucleus. *C*, representative TEM images of sperm cross sections from *Lrrc71*^*+/+*^ and *Lrrc71*^*−/−*^ mice. CP, central microtubule; Fs, fiber sheath; M, mitochondrion; MTD, microtubule doublets; ODF, outer dense fiber. Scale bar is 200 nm. *D*, immunofluorescence (IF) staining was performed to visualize mitochondrial sheath (*red color*), SEPTIN4 protein expression (*green color*), and nuclei (*blue color*) in spermatozoa from *Lrrc71*^*+/+*^ and *Lrrc71*^*−/−*^ mice. Scale bar is 10 μm. *E*, percentage of sperm with abnormal midpiece-principal piece connections in *Lrrc71*^*+/+*^ and *Lrrc71*^*−/−*^ mice. At least 200 sperm per mouse were counted (n = 3). Student’s *t* test. *F*, percentage of SEPTIN4-positive sperm in *Lrrc71*^*+/+*^ and *Lrrc71*^*−/−*^ mice. At least 200 sperm per mouse were counted (n = 3). Student’s *t* test. All data are presented as mean ± SD. ns: no significant difference. ∗*p* < 0.05.

To investigate the underlying causes of impaired sperm motility and acrosomal function in *Lrrc71* KO mice, the ultrastructure of epididymal sperm from *Lrrc71* KO mice was assessed through scanning electron microscopy (SEM) and transmission electron microscopy (TEM). SEM results showed the abnormal midpiece-principal piece junction (Fig. 3*A*), which is consistent with the abnormal sperm tail structure observed in Figure 2*F*. TEM analyses revealed the detachment between the inner acrosomal membrane and the nuclear envelope in *Lrrc71* KO sperm (Fig. 3*B*). Meanwhile, partial loss of the mitochondrial sheath was observed at the junction between the midpiece and principal piece in *Lrrc71* KO sperm, with only outer dense fibers surrounding the axoneme (Fig. 3*B*). Cross-sections of the flagellar axoneme in *Lrrc71* KO sperm displayed abnormal mitochondrial sheaths, although a normal “9 + 2” microtubule organization and normal outer dense fibers were observed (Fig. 3*C*). The sperm annulus is a septin-based structure consisting of multiple septin proteins, which is critical for maintaining the structure of mammalian sperm (24, 25). We performed IF staining with MitoTracker to visualize the mitochondrial sheath, and the anti-SEPTIN4 primary antibody to mark the annulus. Results showed a remarkedly increased distance between the mitochondrial sheath and the annulus in *Lrrc71* KO sperm (Fig. 3*D*). Although the percentage of SEPTIN4-positive sperm in WT and *Lrrc71* KO mice was comparable (Fig. 3*F*), the percentage of sperm with the defective mitochondrial sheath was increased significantly in *Lrrc71* KO mice when compared with WT mice (Fig. 3*E*), indicating the mitochondrial sheath defects at the junction between the midpiece and principal piece in *Lrrc71* KO sperm. Taken together, these results reveal that *Lrrc71* deletion in mice causes impairment of sperm structure including acrosome organization and the mitochondrial sheath near the midpiece-principal piece junction.

### Disrupted glycolysis and oxidative phosphorylation in the sperm with *Lrrc71* deletion

**Figure 4:**
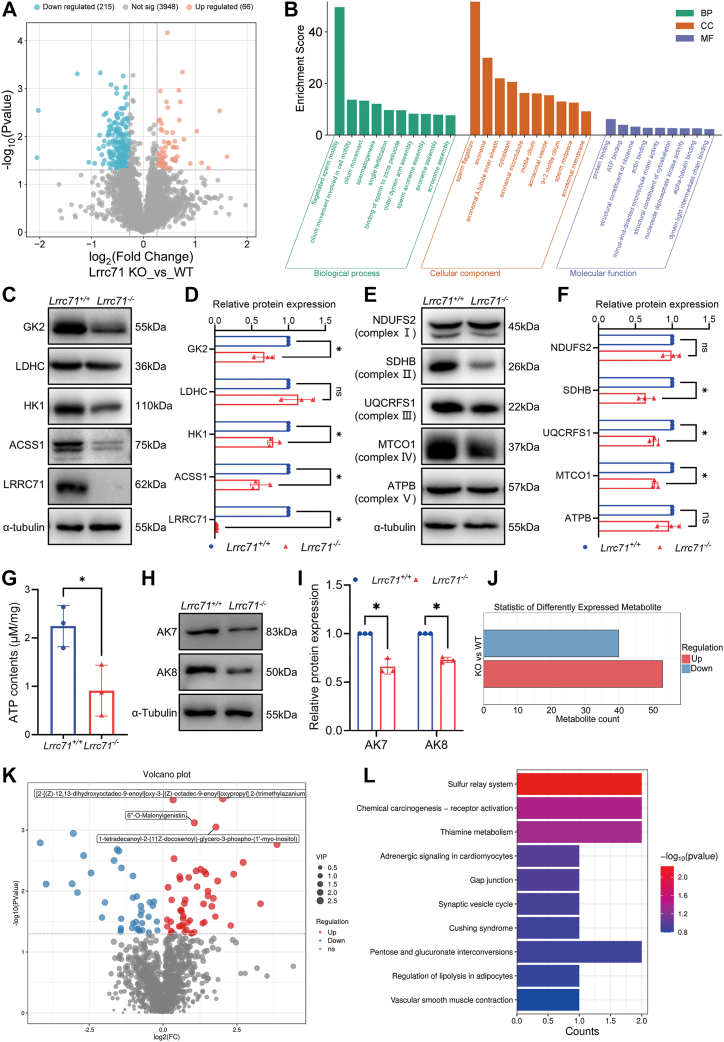
**Proteomic and metabolomic analyses of sperm from *Lrrc71*^*−/−*^ mice.***A*, differentially expressed proteins (DEPs) between sperm from *Lrrc71*^*+/+*^ and *Lrrc71*^*−/−*^ mice. Red dots represent up-regulated proteins, *blue dots* represent down-regulated proteins, *p* < 0.05, |log_2_(Fold change) | >1.2. *B*, *top* 10 significantly enriched Gene Ontology terms of DEPs between sperm from *Lrrc71*^*+/+*^ and *Lrrc71*^*−/−*^ mice. *C*, protein expression of GK2, LDHC, HK1, ACSS1, and LRRC71 in *Lrrc71*^*+/+*^ and *Lrrc71*^*−/−*^ sperm. *D*, quantitation of GK2, LDHC, HK1, ACSS1, and LRRC71 protein expression in sperm (*C*) (n = 3). Mann-Whitney *U* test. ns: no significant difference. ∗*p* < 0.05. *E*, protein expression of NDUFS2, SDHB, UQCRFS1, MTCO1, and ATPB in *Lrrc71*^*+/+*^ and *Lrrc71*^*−/−*^ sperm. *F*, quantitation of NDUFS2, SDHB, UQCRFS1, MTCO1, and ATPB protein expression in sperm (*E*) (n = 3). Mann–Whitney *U* test. ns: no significant difference. ∗*p* < 0.05. *G*, ATP contents in *Lrrc71*^*+/+*^ and *Lrrc71*^*−/−*^ sperm (n = 3). Student’s *t* test. ∗*p* < 0.05. (*H*) Protein expressions of AK7 and AK8 in *Lrrc71*^*+/+*^ and *Lrrc71*^*−/−*^ sperm. *I*, quantitation of AK7 and AK8 protein expression in sperm (*H*) (n = 3). Mann-Whitney *U* test, Student’s *t* test. ∗*p* < 0.05. *J*, number of differentially expressed metabolites in sperm from *Lrrc71*^*+/+*^ and *Lrrc71*^*−/−*^ mice. *K*, differentially expressed metabolites between sperm from *Lrrc71*^*+/+*^ and *Lrrc71*^*−/−*^ mice, with thresholds of VIP > 1 and *p*-value < 0.05. *L*, top 10 significantly enriched pathways for metabolites differing between *Lrrc71*^*+/+*^ and *Lrrc71*^*−/−*^ sperm. All data are presented as mean ± SD.

To further explore the potential mechanisms by which *Lrrc71* KO impairs sperm motility and acrosome function, proteomic analysis was performed with the sperm from WT and *Lrrc71* KO mice. A total of 4229 proteins were identified in the mouse sperm, among which 215 proteins were down-regulated significantly and 66 proteins were up-regulated (Fig. 4*A*). Gene Ontology (GO) enrichment analysis showed that these differentially expressed proteins (DEPs) were significantly enriched in sperm flagellum motility, flagellum assembly, sperm flagellum, axoneme, protein binding, and ATP binding (Fig. 4*B*). ATP production is essential for sperm movement, while glycolysis and OXPHOS are two primary pathways for ATP production in mammalian sperm (6, 9, 26). Therefore, glycolysis-related DEPs from proteomic analysis were validated using Western blot. As shown in Figure 4, *C* and *D*, the protein expression of glycerol kinase 2 (GK2), hexokinase 1 (HK1) and acyl-CoA synthetase short-chain family member 1 (ACSS1) was significantly down-regulated in *Lrrc71* KO sperm, while lactate dehydrogenase C (LDHC) protein expression in KO sperm was similar to that in WT sperm. Additionally, we analyzed the protein expression of critical enzymes in OXPHOS complexes and found a significant decrease in succinate dehydrogenase complex iron-sulfur subunit B (SDHB), ubiquinol-cytochrome C reductase rieske iron-sulfur polypeptide 1 (UQCRFS1), and mitochondrially encoded cytochrome C oxidase I (MTCO1) protein levels in *Lrrc71* KO sperm, as well as unchanged expression of NADH dehydrogenase iron-sulfur protein 2 (NDUFS2) and ATP synthase subunit beta (ATPB) (Fig. 4, *E* and *F*). The down-regulation of glycolysis-or OXPHOS-related proteins in *Lrrc71* KO sperm implies a disruption of ATP production. As hypothesized, a markedly ATP content reduction was detected in *Lrrc71* KO sperm (Fig. 4*G*). Given that sperm movement is driven by ATP hydrolysis *via* adenylate kinase (AK), which provides energy for microtubule sliding and movement, we further analyzed the expression of AK enzymes in *Lrrc71* KO sperm. In accordance with the proteomic data, AK7 and AK8 were down-regulated significantly following *Lrrc71* deletion (Fig. 4, *H* and *I*), which suggests an impaired ATP hydrolysis in *Lrrc71* KO sperm.

To further elucidate the underlying causes of impaired sperm motility, we performed non-targeted metabolomic analysis of sperm samples. A total of 1302 metabolites were identified, among which 53 metabolites were significantly enriched, and 40 metabolites were significantly diminished in abundance in *Lrrc71* KO sperm (Fig. 4, *J* and *K*). KEGG enrichment analysis revealed that the differentially accumulated metabolites were predominantly enriched in sulfur relay system and thiamine metabolism (Fig. 4*L*). Crucially, two differentially accumulated metabolites, namely L-cysteine and thiamine (vitamin B1), were included in sulfur relay system and thiamine metabolism, and were both diminished significantly following *Lrrc71* deletion, suggesting that they are key metabolites regulated by LRRC71 in sperm metabolism. As critical metabolic regulators, L-cysteine maintains cellular redox balance and respiratory chain integrity (27, 28, 29), while thiamine functions as a key coenzyme for core carbohydrate metabolism (30); both metabolites are indispensable for sustaining efficient glycolysis and mitochondrial OXPHOS, thereby ensuring sufficient ATP supply in sperm. Collectively, our results demonstrate that LRRC71 plays essential roles in glycolysis and OXPHOS for ATP production in mouse sperm.

### Impaired fertilization and sperm migration into the oviduct in *Lrrc71*-deficient mice

**Figure 5:**
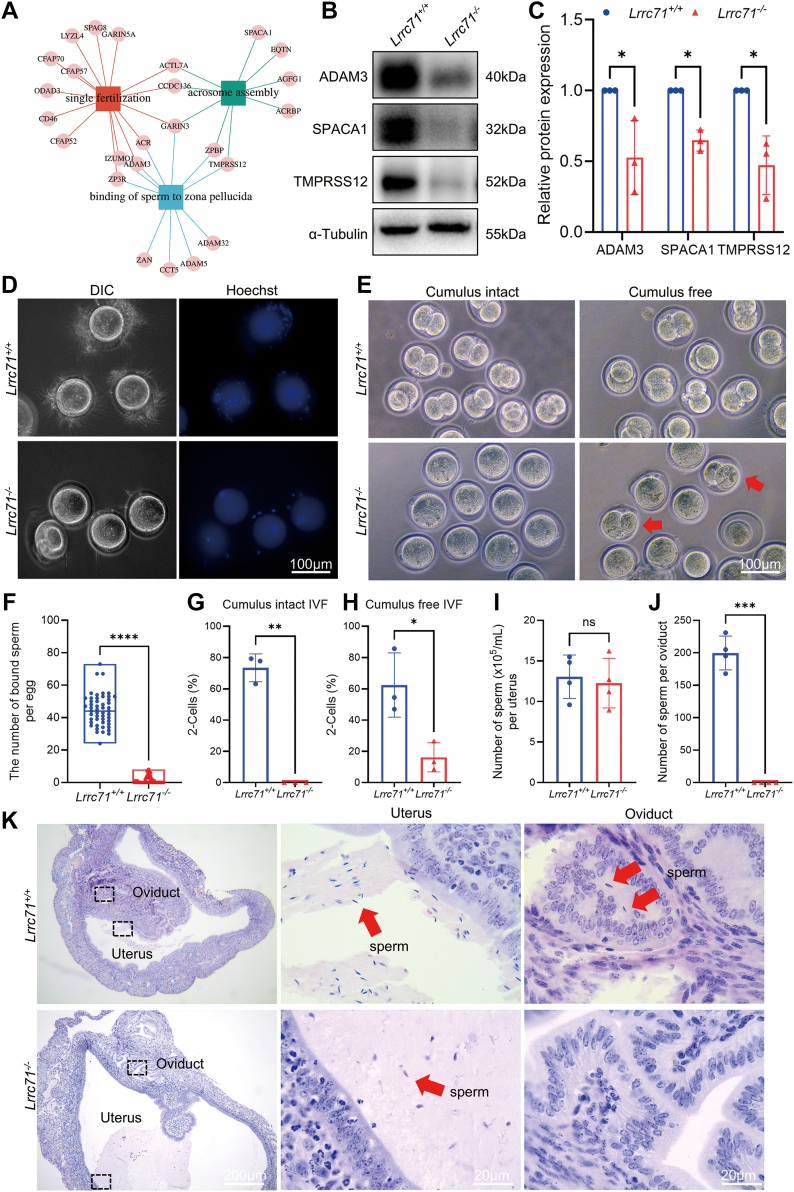
**Assessment of fertilization and oviduct migration of sperm from *Lrrc71*^*−/−*^ mice.***A*, differentially expressed proteins (DEPs) of sperm that were enriched in the biological process of “single fertilization”. *B*, protein expression of ADAM3, SPACA1 and TMPRSS12 in sperm from *Lrrc71*^*+/+*^ and *Lrrc71*^*−/−*^ mice. *C*, quantitation of ADAM3, SPACA1 and TMPRSS12 protein expression in sperm (*B*) (n = 3). Mann-Whitney *U* test. ∗*p* < 0.05. *D*, *in vitro* fertilization (IVF) with epididymal sperm from adult *Lrrc71*^*+/+*^ and *Lrrc71*^*−/−*^ mice. Sperm heads were stained with Hoechst 33,342. Scale bar is 50 μm. *E*, representative images of embryos derived from *Lrrc71*^*+/+*^ and *Lrrc71*^*−/−*^ sperm 24 h post IVF. Cumulus-intact or cumulus-free oocytes were fertilized with epididymal sperm from adult *Lrrc71*^*+/+*^ or *Lrrc71*^*−/−*^ mice. Scale bar is 50 μm. *F*, number of bound sperm per egg for *Lrrc71*^*+/+*^ and *Lrrc71*^*−/−*^ sperm. Student’s *t* test. ∗∗∗∗*p* < 0.0001. *G*, the percentage of 2-cell rate after fertilization of cumulus intact oocytes with epididymal sperm from adult *Lrrc71*^*+/+*^ or *Lrrc71*^*−/−*^ mice (n = 3). Student’s *t* test. ∗∗*p* < 0.01. (*H*) The percentage of 2-cell rate after fertilization of cumulus free oocytes with epididymal sperm from adult *Lrrc71*^*+/+*^ or *Lrrc71*^*−/−*^ mice (n = 3). Student’s *t* test. ∗*p* < 0.05. *I*, sperm counts in the uterus after coitus (*n* = 4). Student’s *t* test. ns: no significant difference. *J*, sperm counts in the oviducts after coitus (*n* = 4). Student’s *t* test. ∗∗∗*p* < 0.001. *K*, H&E staining of utero-tubal junction cross sections from females mated with *Lrrc71*^*+/+*^ or *Lrrc71*^*−/−*^ males. The middle and right panels show the higher-magnification views of boxed areas in the *left panels*, with sperm indicated by *red arrows*. Scale bar is 200 μm or 20 μm. All data are presented as mean ± SD.

In addition to sperm motility, DEPs in *Lrrc71* KO sperm were enriched in acrosome assembly, fertilization, and sperm–zona pellucida (ZP) binding (Fig. 5*A*), implying the impact of *Lrrc71* deletion on fertilization. Western blot analyses of proteins involved in these processes revealed that a disintegrin and metallopeptidase domain 3 (ADAM3), sperm acrosome-associated 1 (SPACA1), and transmembrane protease, serine 12 (TMPRSS12) protein levels were decreased significantly in *Lrrc71* KO sperm (Fig. 5, *B* and *C*). Furthermore, the binding ability of *Lrrc71* KO sperm to eggs was markedly reduced (Fig. 5, *D* and *F*). *In vitro* fertilization (IVF) assays showed that *Lrrc71* KO sperm completely failed to fertilize cumulus-intact oocytes (Fig. 5, *E* and *G*). Although the fertilization was partially restored after cumulus cell removal, the fertilization rate of *Lrrc71* KO sperm was still significantly lower than that of WT sperm (Fig. 5, *E* and *H*). These results indicate that *Lrrc71* deletion in mice causes defective sperm fertilization.

Previous studies have reported that ADAM3 is critical for normal sperm migration within the female reproductive tract (31, 32). Due to the decrease in ADAM3 level in *Lrrc71* KO sperm, we assessed the effect of *Lrrc71* deletion on sperm migration in the uterus and oviduct. Although *Lrrc71* KO males mated normally with the WT females and total uterine sperm counts were comparable between WT and *Lrrc71* KO groups (Fig. 5*I*), no sperm was detected in the oviducts of females mated with *Lrrc71* KO males (Fig. 5*J*). Histological examination confirmed the presence of sperm in both the uterus and oviduct of females mated with WT males, whereas sperm was observed only in the uterus and not in the oviduct of females mated with KO males (Fig. 5*K*). These results indicate that *Lrrc71* deletion in mice impaired sperm migration into the oviduct. Together, our findings demonstrate that LRRC71 deficiency not only reduces sperm motility, but also impairs fertilization capacity and sperm migration into the oviduct.

### LRRC71 interacts with proteins regulating sperm motility and fertilization

**Figure 6:**
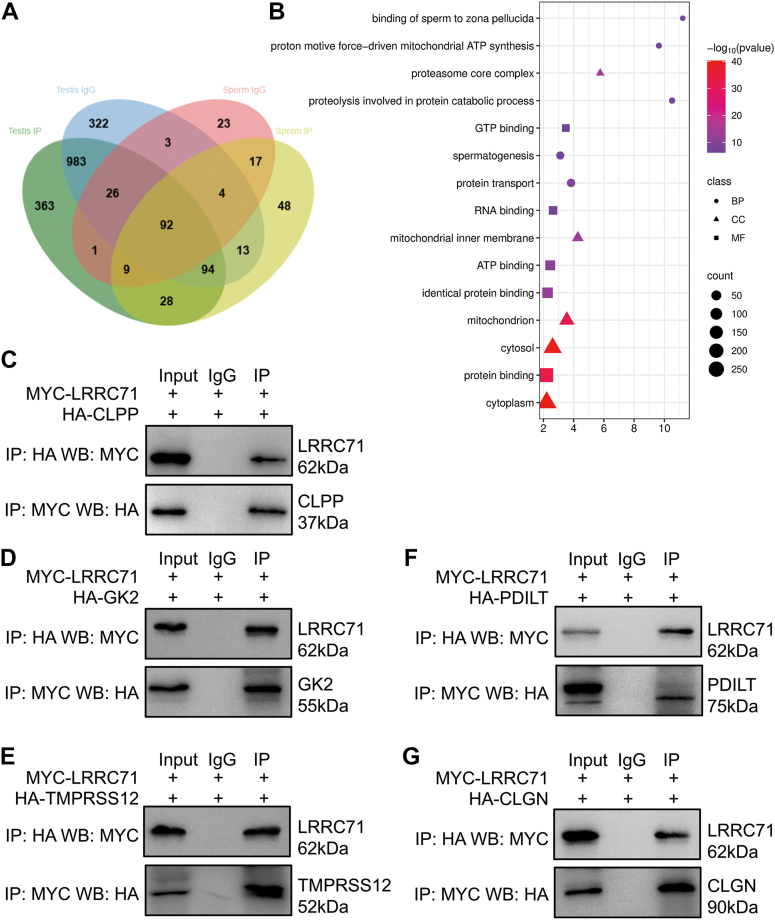
**Identification of LRRC71-interacting proteins in the testes and sperm of mice.***A*, Venn diagram illustrating the number of LRRC71-binding proteins in mouse testicular tissue and sperm lysates. *B*, *top* five significantly enriched Gene Ontology terms for LRRC71-binding proteins in mouse testicular tissue and sperm lysates. *C–G*, co-immunoprecipitation was performed to validate the interactions between LRRC71 and CLPP, GK2, TMPRSS12, CLGN, PDILT in HEK-293T cells.

To explore the molecular mechanisms by which *Lrrc71* deletion leads to reduced sperm motility and impaired fertilization, immunoprecipitation followed by mass spectrometry (IP-MS) was performed to identify the interacting partners of LRRC71 in mouse testes and sperm. A total of 401 and 183 interacting proteins were identified in testes and sperm, respectively (Fig. 6*A*). GO enrichment analysis revealed that these interacting proteins were primarily involved in binding of sperm to zona pellucida, proton motive force-driven mitochondrial ATP synthesis, GTP binding and spermatogenesis (Fig. 6*B*). Caseinolytic mitochondrial matrix peptidase proteolytic subunit (CLPP) and GK2 are potential LRRC71-binding partners involved in proton motive force-driven mitochondrial ATP synthesis process. Co-IP experiments validated their interactions with LRRC71 *in vitro* (Fig. 6, *C* and *D*). TMPRSS12, protein disulfide isomerase-like, testis-expressed (PDILT) and calmegin (CLGN) are included in the biological process of binding of sperm to zona pellucida. The interactions between LRRC71 and TMPRSS12, PDILT, and CLGN were also validated *in vitro* (Fig. 6, *E*–*G*). Collectively, these results suggest that LRRC71 could form a functional protein complex with proteins regulating sperm motility and fertilization, which affects the expression of these proteins, leading to reduced sperm motility and impaired fertilization capacity.

### Identification of *LRRC71* variants in humans with asthenozoospermia

**Figure 7:**
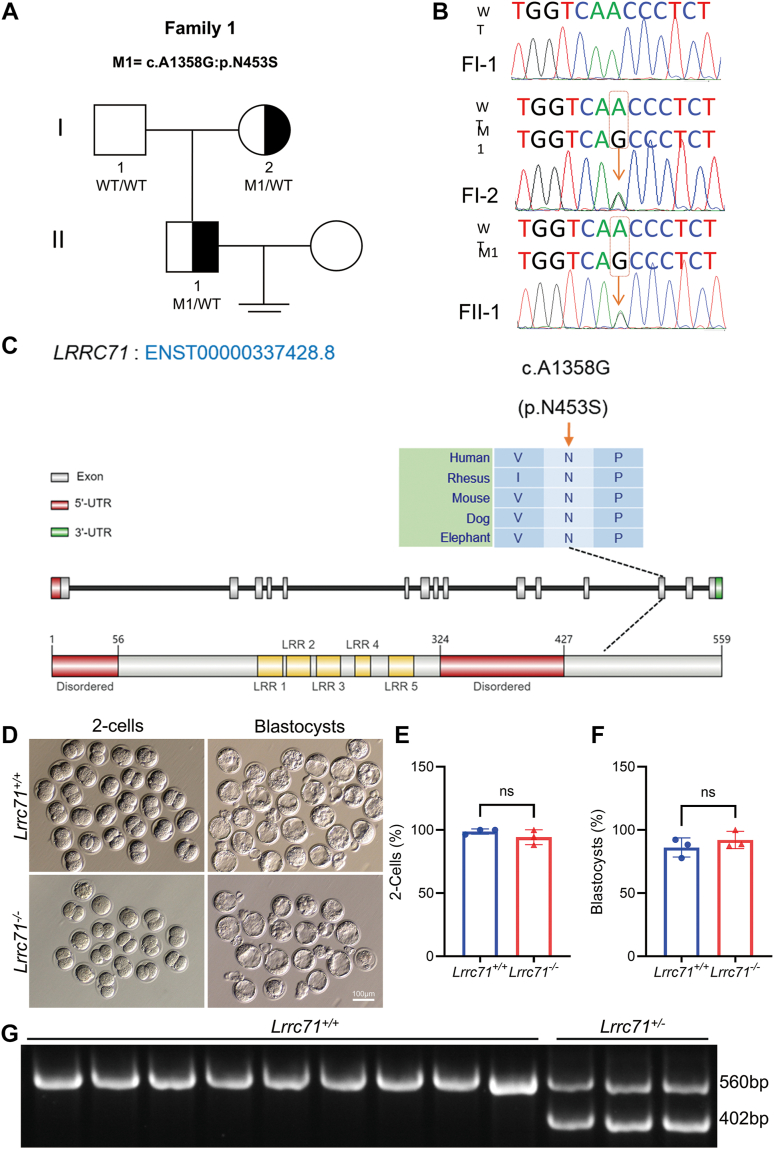
**Identification of pathogenic *LRRC71* variants in infertile males.***A*, pedigree of a family carrying pathogenic *LRRC71* variants identified by whole-exome sequencing (WES). Filled symbols indicate individuals carrying the variants. Squares represent male and circles represent female family members. *B*, Sanger sequencing validation of the *LRRC71* c.1358A>G variant. The proband (II-1) and his mother (I-2) are heterozygous carriers, with the mutant base indicated by an orange arrow. *C*, schematic depicting the *LRRC71* protein domain structure, with the disease-associated variant (c.1358A>G) indicated by a black dotted line. *D*, representative bright-field images of 2-cell and blastocyst stage embryos following intracytoplasmic sperm injection (ICSI) using sperm from *Lrrc71*^*+/+*^ or *Lrrc71*^*−/−*^ mice. Scale bar is 100 μm. Quantitative analyses of the rates of 2-cell embryo formation (*E*) and blastocyst development. *F*, Student’s *t* test. Data are presented as mean ± SD. ns: no significant difference. *G*, PCR genotyping results of newborn mice conceived *via* ICSI. Band size: wild-type (*Lrrc71*^+/+^, 560 bp), heterozygous (*Lrrc71*^+/−^, 560/402 bp).

To investigate the potential involvement of *LRRC71* variants in human male infertility, we screened a cohort of 725 patients with asthenoteratospermia and 400 fertile males as controls using whole-exome sequencing (WES). A heterozygous missense mutation in *LRRC71* (c.A1358G) was identified in one individual. This variant exhibited an extremely low minor allele frequency in the 1KGP, ExAC, and gnomAD databases, and it was predicted to be damaging or pathogenic by SIFT, Polyphen-2, and MutationTaster (Table 1). Sanger sequencing confirmed that this variant was inherited from a heterozygous parent carrier (I-2) (Fig. 7, *A* and *B*). The LRRC71 protein is highly conserved and consists of 559 amino acids; the identified mutation (p.N453S) is located within a conserved domain (Fig. 7*C*). Semen analysis showed that the sperm count of this patient was within the normal range, but the total motility and progressive motility of sperm were both reduced markedly (Table 2). These findings suggest that *LRRC71* is a potential pathogenic gene of infertile humans with asthenoteratospermia.

**Table 1:** Genetic characterization of *LRRC71* variant of the patient

Subject	Patient
cDNA mutation	c.A1358G
Exon	Exon13
Mutation type	Missense
Protein alteration	p.N453S
Allele frequency in human population	
1KGP_all	-
ExAC_all	0.00002436
gnomAD_exome_all	0.000009067
Deleterious prediction	
SIFT	D
Polyphen2_HVAR	D
CADD_PHRED	19.72

RefSeq accession number of *LRRC71* is NM_144702.3.

1KGP_all, all the data of 1000 Genomes Project; CADD, Combined Annotation Dependent Depletion; D, disease-causing; ExAC_all, all the data of Exome Aggregation Consortium; gnomAD_exome_all, overall allele frequency in the Genome Aggregation Database exome release v2.1.1, combining all population groups; SIFT, Sorting Intolerant From Tolerant.

**Table 2:** Semen parameters in the patient carrying heterozygous *LRRC71* variant

Subject	Patient	Reference values^a^
Age	40	
Semen parameters		
Semen volume (mL)	2.3	>1.5
Concentration (10^6^/ml)	22.4	>15.0
Motility (%)	6.4	>40.0
Progressive motility (%)	3.2	>32.0

a Reference Values according to the WHO (2010) criteria.

To investigate whether intracytoplasmic sperm injection (ICSI) can be a potential approach for treating infertile humans harboring *LRRC71* mutations, we attempted to rescue male infertility of mice caused by *Lrrc71* deletion using ICSI. The rates of 2-cell embryo development and blastocyst formation in the *Lrrc71* KO groups after ICSI showed no statistically significant difference compared with those in the WT group (Fig. 7, *D*–*F* and Table S3). Transferring the blastocysts in the *Lrrc71* KO groups into surrogate females yielded a total of nine WT and three heterozygous offspring (Fig. 7*G*). Our results suggest that ICSI is an effective approach for treating *LRRC71*-related asthenoteratospermia.

## Discussion

The LRRC protein family comprises hundreds of members characterized by leucine-rich repeat motifs that play diverse roles in multiple biological processes. Notably, accumulating studies have reported the critical roles of LRRC family members in spermatogenesis and male fertility (14, 16, 17, 33). Nevertheless, the function of LRRC71 in mammalian spermatogenesis and male fertility is still unknown. Our study demonstrates that LRRC71 is essential for mouse spermiogenesis and male fertility. Meanwhile, a novel heterozygous *LRRC71* mutation was identified in a sterile patient with asthenozoospermia. These findings uncover a novel role of LRRC71 in spermatogenesis and a potential target for the diagnosis and treatment of male asthenozoospermia.

During spermiogenesis, from round spermatids to mature sperm, the mitochondria of germ cells transform from a classic structure to a more condensed structure, implying their critical roles in sperm functions (34). It has been reported that defective structure of mitochondria in the midpiece of sperm influences sperm motility and male fertility (9, 35). In our results, the abnormal ultrastructure of mitochondrial sheaths in *Lrrc71* KO mice was observed, which may account for the reduced sperm motility. Similarly, knockout of other LRRC family members also causes impaired mitochondrial sheaths, reduced sperm motility, and male infertility, such as *Lrrc8* (36), *Lrrc46* (33), and *Lrrc56* (37). Moreover, incomplete mitochondrial sheath in the midpiece of sperm was observed in an asthenozoospermia patient carrying a *LRRC6* homozygous mutation (14). These findings reveal the important roles of LRRC family members in the assembly of mitochondrial sheath and the acquisition of sperm motility. Although deletion of *Lrrc71* or other *Lrrc* genes in mice induced defective mitochondrial sheath structure, the resulting phenotypic defects were different. *Lrrc8*, *Lrrc46*, and *Lrrc56* KO male mice exhibit abnormal sperm morphology including tailless, shortened tails, and coiled tails, accompanied by condensed or disorganized mitochondrial sheaths (33, 36, 37). By contrast, in *Lrrc71* KO mice, the major structural defects were in the midpiece of the sperm tail. IF staining results showed that the structural defects occurred near the annulus, where the midpiece and the principal piece connects in the sperm tail. TEM results revealed that the structural defects were caused by the loss of mitochondrial sheath in the terminal region of the sperm midpiece. Unlike deficiencies of other LRRC family members that induce structural defects in sperm flagella, *Lrrc71* deletion caused abnormal mitochondrial sheath in mouse sperm, indicating distinct molecular mechanisms underlying the involvement of LRRC family members in sperm morphogenesis. Meanwhile, these findings also emphasize the unique effect of *Lrrc71* deletion on mitochondrial sheath assembly. We proved that LRRC71 interacted with GK2, a key regulator of mitochondrial sheath assembly and function. GK2 interacts with several critical mitochondrial and spermiogenesis-associated proteins (38), which suggests that LRRC71 could form a protein complex with other proteins including GK2 to regulate the assembly and homeostasis of mitochondrial sheath in sperm.

As a “power plant” of a cell, mitochondria play a central role in producing ATP to support cellular biological reactions. The mitochondrial sheath in the midpiece of sperm tail produces ATP to supply the energy for sperm movement (9, 39). Due to the impaired structure of mitochondrial sheaths and reduced sperm motility in *Lrrc71* KO mice, we further assessed the ATP production in sperm. As hypothesized, the ATP content was decreased significantly in *Lrrc71* KO sperm when compared with the WT group. There are two main ways of producing ATP in sperm: glycolysis and OXPHOS (40, 41). We found that LRRC71 deficiency affected both sperm metabolism pathways. GK2 and HK1 are key enzymes closely related to glycolysis in sperm metabolism. *Gk2* KO male mice exhibited reduced sperm motility and infertility due to the disorganized mitochondrial sheath formation and aberrant ATP production (38, 42). Although the sperm motility and ATP production were normal in *Hk1* KO male mice, the sperm glycolytic metabolism was suppressed (43). The significantly decreased protein expression of GK2 and HK1 was detected in sperm of *Lrrc71* KO mice, indicating a disruption of glycolysis in sperm after LRRC71 deficiency. SDHB (44), UQCRFS1 (45) and MTCO1 (46) are all key components of OXPHOS complexes and have critical functions in ATP production. LRRC71 deficiency induced a significant decrease in SDHB, UQCRFS1 and MTCO1 protein levels, indicating the impairment of OXPHOS complexes in the sperm. Collectively, both glycolysis and OXPHOS were disrupted in *Lrrc71* KO sperm, thereby resulting in reduced ATP production.

L-cysteine and thiamine were identified as differential metabolites between WT and *Lrrc71* KO sperm, with significant enrichment in the sulfur relay system and thiamine metabolism pathways. In the hypothermic liquid storage of boar semen, l-cysteine supplementation could improve sperm quality (47, 48), which suggests it has a protective effect on sperm structure and functions. Thiamine is a protectant in the cryopreservation of ram semen (49), and its deficiency in metronidazole-treated rats was closely related to the observed infertility side effects (50). The significant decrease of L-cysteine and thiamine content in *Lrrc71* KO sperm indicates that these two metabolites are targets of LRRC71 during spermatogenesis. Their depletion may impair mitochondrial sheath formation, reduce sperm motility, and compromise other essential sperm functions. Further experiments are required to investigate the roles of l-cysteine and thiamine in spermatogenesis as well as the specific mechanism by which LRRC71 modulates the metabolism of these two components in sperm.

The significantly enriched pathway “single fertilization” was identified in the DEPs between WT and *Lrrc71* KO sperm, suggesting the occurrence of fertilization defects following *Lrrc71* deletion. Our experiments validated defective fertilization and failed migration of sperm into the oviduct in *Lrrc71* KO mice. Previous studies have shown that *Tmprss12* KO or *Adam3* KO male mice exhibited impaired sperm-ZP binding and failed migration of sperm from the uterus to the oviduct (31, 51, 52). Coincidentally, reduced protein levels of TMPRSS12 and ADAM3 were detected in *Lrrc71* KO sperm, suggesting that LRRC71 regulates the fertilization ability of sperm *via* TMPRSS12 and ADAM3. Furthermore, ADAM3 protein expression was disrupted in *Tmprss12* KO sperm, which reveals that ADAM3 may be the downstream of TMPRSS12 (51). These findings suggest that LRRC71 regulates ADAM3 expression through TMPRSS12 in sperm fertilization and oviduct migration. Notably, the impaired migration of *Lrrc71* KO sperm in the female reproductive tract may not be solely attributed to reduced ADAM3 expression, but also closely associated with severely decreased progressive motility. *Lrrc71* deletion causes ∼85% of sperm to lose progressive motility, abolishing their ability to traverse the tract. Among the remaining motile sperm, reduced ADAM3 expression further hinders transit. Thus, the combined defects of impaired motility and abnormal ADAM3 may completely prevent *Lrrc71* KO sperm from traversing the female reproductive tract. Since LRRC71 is highly expressed in the fallopian tube, the preserved fertility of female *Lrrc71*-KO mice is somewhat unexpected, raising the possibility of impaired oviductal ciliary function. Our results showed that the ciliary morphology in the *Lrrc71* deletion oviduct was normal, which reveals the normal function of oviductal ciliary. Due to functional redundancy of LRRC71 in the oviduct (53), the loss of this gene may be functionally compensated by other LRRC family paralogs.

Testis-specific ER chaperones, including PDILT and CLGN, are crucial for ADAM3 maturation (54, 55). These chaperones form a protein complex that folds ADAM3 in the ER and transfers the mature form to the sperm membrane. The interactions between LRRC71 and the chaperones PDILT and CLGN were validated *in vitro*, which suggests that LRRC71 localizes to the ER. Due to the lack of available antibody to LRRC71, the subcellular localization of LRRC71 was investigated by transfecting the EGFP-LRRC71 fusion protein into different cells. Results showed that EGFP-LRRC71 exhibited a predominantly diffuse cytoplasmic distribution, which was inconsistent with its predicted ER localization. This may be attributed to the differences between *in vivo* and *in vitro* experiments. LRRC71 deficiency may disrupt the function of chaperones, which affects the correct folding of ADAM3, and thus results in impaired sperm-ZP binding and oviduct migration. Additionally, LRRC71, CLGN and ADAM3 are all involved in protein trafficking of sperm maturation. As protein trafficking functions upstream of nearly all physiological processes in spermatozoa, ablation of a single trafficking-related protein often leads to multiple downstream defects. This consequently complicates the identification of the gene product’s precise molecular function, as well as its relative position within the regulatory hierarchy. Therefore, the exact function of LRRC71 in protein folding and trafficking needs further investigation.

We not only demonstrated the low sperm motility and male infertility in *Lrrc71* KO mice but also identified a novel *LRRC71* heterozygous mutation in an infertile clinical patient with asthenozoospermia. Studies have shown that IVF and ICSI represent potential solutions for the treatment of patients with asthenozoospermia (56, 57). We found that *Lrrc71* KO sperm could not fertilize the oocytes with intact cumulus successfully. Following cumulus cell removal, a slight increase in fertilization rate was observed in *Lrrc71* KO sperm, yet the fertilization capacity was still functionally impaired. These findings imply an elevated risk of IVF-based assisted reproductive treatments (ARTs) for patients with asthenozoospermia harboring *LRRC71* mutations. It has been reported that clinical ICSI treatment in asthenozoospermia infertile patients carrying *LRRC23* or *LRRC6* homozygous mutations resulted in successful pregnancies (14, 58), which suggests that ICSI may be effective for *LRRC71*-mutant patients. Therefore, we assessed the ICSI outcomes of *Lrrc71* KO male mice. Strikingly, the formation rates of two-cell embryos and blastocysts were comparable between WT and *Lrrc71* KO sperm, as well as the successful production of offspring in the KO groups. Collectively, our findings reveal that ICSI may be a promising and effective approach for treating patients with asthenozoospermia carrying *LRRC71* mutations.

**Figure 8:**
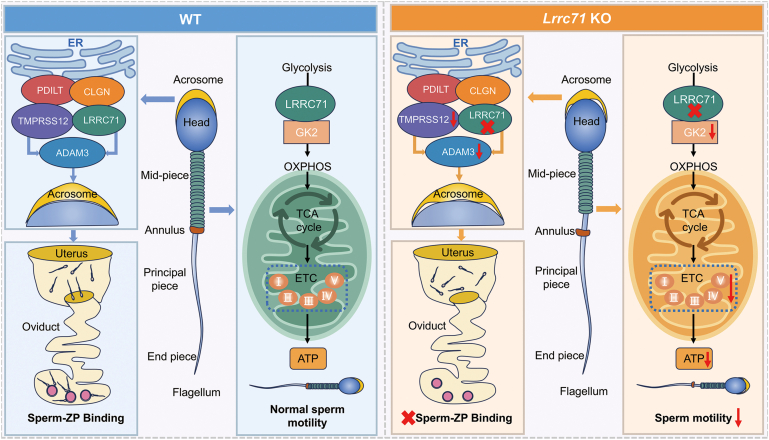
**Diagram of proposed action of *Lrrc71* in mouse spermatogenesis.** Diagram of proposed action of *Lrrc71*. Deletion of *Lrrc71* severely impairs sperm motility through disruption of energy metabolism pathways, including glycolysis and oxidative phosphorylation (OXPHOS). Concurrently, LRRC71 interacts with endoplasmic reticulum chaperones to ensure proper ADAM3 maturation, and its absence leads to defective sperm migration into the oviduct and impaired fertilization. This figure was originally created by the authors using PowerPoint.

Elucidating the underlying pathogenic mechanisms of *Lrrc71* deletion will be of great help for us to understand the complex processes of sperm morphogenesis and sperm maturation. We show that *Lrrc71* is required for mitochondrial sheath assembly, sperm motility, fertilization, and oviduct migration in mice. Both glycolysis and OXPHOS were damaged in *Lrrc71* KO sperm, resulting in reduced ATP production. LRRC71 may disrupt ADAM3 maturation by interacting with ER chaperones, which in turn causes defective fertilization and impaired sperm migration into the oviduct (Fig. 8). Taken together, we identify LRRC71 as an essential regulator of sperm motility, fertilization and oviduct migration, representing a potential target for both the diagnosis and treatment of infertile patients with asthenozoospermia and the development of male contraceptive drugs.

## Experimental procedures

### Ethics statement

The Institutional Animal Care and Use Committee of Yangzhou University examined and approved all experiments involving live animals (Approval No. YXYLL-2023-049). The Guide for the Care and Use of Laboratory Animals was followed during the experiment. The First Affiliated Hospital of Anhui Medical University authorized the human investigations (Approval No. PJ2023-04-19), and each subject provided written informed consent. The studies in this work abide by the Declaration of Helsinki principles.

### Phylogenetic analysis

Orthologs of LRRC71 were retrieved from the National Center for Biotechnology Information (NCBI) Orthologs. The sequence alignment was performed using the MEGA12 software with the MUSCLE method, and the *p* distance < 0.7. The phylogenetic tree of LRRC71 proteins from various species was constructed using the neighbor-joining method with a bootstrap of 1000 by MEGA12 software.

### Cell culture and transfection

HeLa, NIH3T3, GC1-spg, and GC2-spd cells were obtained from preserved in the lab. HeLa cells were cultured in MEM supplemented with 10% fetal bovine serum (FBS) and 1% penicillin-streptomycin. GC1-spg, GC2-spd and NIH3T3 cells were cultured in DMEM with 10% FBS and 1% penicillin-streptomycin. All cells were maintained at 37 °C in 5% CO_2_. The EGFP-LRRC71 plasmid was purchased from Unibio. Transient transfection was performed using Exfect (Vazyme) according to the manufacturer’s protocol. At 24 h post-transfection, cells were fixed with 4% paraformaldehyde for 5 min and stained with Hoechst 33,342 (1 μg/ml). Organelle-specific probes were used: MitoTracker Red CMXRos (200 nM, Beyotime) for mitochondria, ER-Tracker Red (1 μm, Beyotime) for ER, and LysoTracker Red (50 nM, Beyotime) for lysosomes. Images were acquired using a confocal microscope (Nikon Ti2).

### Generation of *Lrrc71* knockout mouse model

GemPharmatech supplied the *Lrrc71* knockout mice (strain number T045404). In short, gRNAs were transcribed *in vitro*. The fertilized eggs of C57BL/6JGpt mice were microinjected with Cas9 and gRNAs. Positive F0 mice were produced by transplanting fertilized eggs, and this was verified by PCR and on-target amplicon sequencing. By mating positive F0-generation mice with C57BL/6JGpt animals, a stable F1-generation mouse strain was produced. PCR and on-target amplicon sequencing were used to validate the intended mutant allele.

### RNA isolation, RT-PCR

Tissues were treated with TRIzol Reagent (Invitrogen) to extract total RNA. Using a cDNA Reverse Transcription Kit (Takara), 1 μg of RNA from each specimen was then reverse-transcribed into cDNA. PCR amplification was carried out with a GeneAmp PCR Systen 9700. The primer sequences are shown in Table S2.

### Fertility assessment

For the male fertility test, six adult *Lrrc71* KO males and six adult WT males were each paired with two WT C57BL/6N females for over two months. For the female fertility test, six adult *Lrrc71* KO females and six adult WT females were each paired with one WT C57BL/6N male for over two months. All parturition events were recorded, including timing and litter size. Fertility was assessed by comparing the average litter size.

### Sperm motility assay

Male WT and *Lrrc71* KO mice were sedated with isoflurane and killed *via* cervical dislocation. After the collection of epididymal sperm, the sperm were placed in human tubal fluid (HTF) culture medium. Sperm motility was assessed using the CEROS II computer-aided sperm analysis system at two time points: 10 min after sperm release (before capacitation) and 2 h after release (after capacitation). For each time point and each sample, approximately 200 sperm were analyzed.

### Histological analysis

Testes from mice were collected and preserved in 4% paraformaldehyde for a full day. Tissues were fixed in paraffin and sectioned at 4 μm onto glass slides after being dehydrated using a graded ethanol series. Deparaffinized slices were stained with either periodic acid-Schiff (PAS) or hematoxylin and eosin (H&E) for histological analysis.

H&E staining was performed by incubating sections with hematoxylin (Beyotime) for 10 min, followed by differentiation in 1% acid-alcohol and rinsing. Subsequent eosin staining lasted 5 min. Sections were then dehydrated through graded alcohols and cleared in xylene before microscopic observation (Nikon-80i).

PAS staining was conducted using a commercial Schiff’s kit (Sbjbio) per manufacturer's instructions. Briefly, slides were treated with periodic acid, rinsed, and incubated with Schiff’s reagent in the dark. Nuclei were counterstained with hematoxylin. Following dehydration and xylene clearing, images were acquired under the same light microscope.

### Scanning electron microscopy

After being liberated from the epididymis, spermatozoa were placed in HTF medium and incubated for 15 min at 37 °C. Following two rounds of washing with phosphate-buffered saline (PBS) and an overnight fixation in 2.5% glutaraldehyde, the suspension was centrifuged at 500*g* for five minutes. After adjusting the sperm density to the proper concentration, a coupling agent was used to adhere the cells to glass slides. Samples were then dried, sputter-coated with gold, and dehydrated using a graded ethanol series. A GeminiSEM 300 scanning electron microscope was used to collect and examine the images.

### Transmission electron microscopy

In 2.5% (v/v) glutaraldehyde made in 0.1 M cacodylate buffer (Sigma), sperm samples from both WT and *Lrrc71* KO mice were frozen for a whole night at 4 °C. The samples were cleaned and then submerged in 1% osmium tetroxide for one hour at 4 °C. Following dehydration using a graded acetone series (50–100%), they were implanted in resin (DDSA, NMA, enhancer, 812 resin (Sigma)). After preparing ultrathin slices (60 nm) and double-staining them with uranyl acetate and lead citrate, an HT7800 transmission electron microscopy was used to capture and analyze the images.

### Preparation of testicular cells and immunofluorescence staining

Adult mice's testes were removed and placed in a 15 ml centrifuge tube with 5 ml of DMEM and 0.5 mg/ml collagenase IV and 1.0 μg/ml DNase I (Sigma). The tissue was centrifuged for five min at 1000 rpm after being incubated for 32 min at 32 °C. The resultant pellet was given a single PBS wash after the supernatant was discarded. The pellet was then distributed onto positively charged glass slides, reconstituted in PBS, and fixed with 4% paraformaldehyde.

Sodium citrate buffer was used for antigen retrieval before IF staining of paraffin-embedded sections. HeLa cells were first fixed with 4% paraformaldehyde for cell-based IF, and then they were permeabilized with 0.3% Triton X-100. Before being incubated overnight at 4 °C with the proper primary antibodies (see Table S1), all samples were blocked with 1% bovine serum albumin. After that, matching secondary antibodies were incubated for an hour at room temperature. Ultimately, pictures were taken right away utilizing a Carl Zeiss Scope AI microscope and a Nikon AXR confocal microscope.

### Western blot

RIPA lysis buffer enhanced with an EDTA-Free Protease Inhibitor Cocktail from MCE was used to extract proteins. Using a BCA protein assay kit (NCM Biotech), the amount of protein was determined. After being separated using SDS-PAGE, the proteins were electrotransferred onto membranes made of polyvinylidene fluoride. Following blocking with 5% non-fat milk, primary antibodies (Table S1) were used to probe the membranes overnight at 4 °C. Horseradish peroxidase-conjugated secondary antibodies were then incubated for 1.5 h at room temperature. An ECL detection system (NCM Biotech) was used to see protein bands, and a Tanon imaging system (5200, Tanon) was used to take pictures. Using Image J software (version 1.47v), band intensity was measured.

### ATP content detection

Before being resuspended in lysis solution and briefly vortexed, spermatozoa were washed twice with buffer and then placed on ice. A commercial ATP Assay Kit (Beyotime) and a luminometer (TD-20/20, Turner Designs) were used to measure intracellular ATP levels. A BCA protein assay was used to calculate the total protein concentration, which was used to normalize the observed ATP content.

### Intracytoplasmic sperm injection

Initially, 5 IU of pregnant mare serum gonadotropin (PMSG) were injected intraperitoneally into four-week-old female mice. Five IU of human chorionic gonadotropin (hCG) were administered intraperitoneally to the mice after 48 h. Thirteen to 15 hours after the delivery of hCG, cumulus-oocyte complexes were extracted from the oviductal ampulla. Male mice's caudal epididymis was used to separate sperm heads, which were then microinjected into the oocytes for ICSI. After that, the injected oocytes were cultivated for 24 h at 37 °C in CBZ medium. After 24 and 96 h of culture, respectively, the development of the embryo to the 2-cell and blastocyst stages was assessed. Ultimately, recipient females received the resultant blastocysts.

### Sperm-egg binding and *in vitro* fertilization

For female mice, the superovulation regimen was the same as it was for ICSI treatments. Cumulus cells were removed from cumulus-oocyte complexes (COCs) by treating them with 330 μg/ml hyaluronidase after they were extracted from the oviductal ampulla. Denuded oocytes were co-incubated with capacitated sperm for 30 min in order to perform sperm-egg binding tests. After fixing sperm-egg complexes in HTF medium drops containing 0.25% glutaraldehyde for 15 min on ice, bound sperm were seen using Hoechst staining. A Zeiss Axio Vert A1 microscope was used for imaging (Carl Zeiss). IVF was carried out by putting both cumulus-free and cumulus-intact oocytes into HTF medium that included sperm (1 × 10^6^/ml). The eggs were cultivated in KSOM medium (Aibei Biotechnology) at 37 °C with 5% CO2 after being incubated for four to six hours and then cleaned with HTF drops. Twenty-four hours following insemination, the rate of 2-cell embryo development was assessed.

### Acrosome reaction analysis

Sperm were extracted from the cauda epididymis of mature male mice and cultured in HTF medium (Aibei Biotechnology) for 90 minutes in order to induce capacitation. The calcium ionophore A23187 was applied to capacitated sperm at a final concentration of 10 μm to trigger the acrosome response. MitoTracker (Beyotime) was used to assess sperm vitality after a 30-min incubation period. Only MitoTracker-positive sperm were then subjected to PNA (Sigma) staining to assess acrosomal status.

### Sperm migration in the female reproductive tract

Oviductal sperm counts and histological analysis of the uterine-tubal junction (UTJ) were used to quantify the migration of sperm from the uterus to the oviduct. Superovulation was used to couple age-matched male mice (WT and *Lrrc71* KO) with eight-week-old female mice. A vaginal plug served as proof of mating. Uterine and oviductal tissues were extracted three hours after coitus, flushed with HTF medium, and centrifuged to extract sperm for counting. 4% paraformaldehyde was used to fix oviduct-UTJ complexes for histological examination. H&E staining was then applied to the sections for microscopic examination.

### Immunoprecipitation-mass spectrometry

Protein was extracted from adult mouse testes and sperm using 1% NP40 lysis buffer. To prepare 10 mg of testicular protein for IP-MS, it was first incubated with 100 μl Protein A/G Agarose (MCE) at 4 °C for one to three hours, and then it was centrifuged at 300*g* for five minutes at 4 °C. The resultant supernatant was incubated with primary antibodies at 4 °C for an entire night while being gently stirred. After adding 200 μl of Protein A/G Agarose, the mixture was rocked for four hours at 4 °C. After that, the agarose beads were collected and rinsed four or five times using 1% NP40 lysis buffer. The Genechem Company performed the mass spectrometry analysis. Acquired raw data were processed with Proteome Discoverer 2.2 (Thermo Fisher Scientific). Proteins were considered confidently identified when at least one peptide matched with a false discovery rate (FDR) ≤ 0.01. DAVID (https://davidbioinformatics.nih.gov/) was used for GO enrichment analysis, and the web tool https://www.bioinformatics.com.cn was used to create heatmaps for data visualization.

### Co-immunoprecipitation

Unibio provided full-length cDNA. RT-PCR was used to amplify the open reading frame, which was then cloned into pCMV-Myc and pCMV-HA vectors. Lipofectamine 2000 (Invitrogen) was used to transfect plasmids into HEK-293T cells. For co-IP, 1 mg of cell lysate protein was precleared with 10 μl Protein A/G Agarose and then incubated with 20 μl Protein A/G Agarose with either anti-HA or anti-MYC antibody. The beads were resuspended in 50 μl of × loading buffer and boiled for five minutes after being washed with 1% NP40 lysis buffer. Western blotting was used to identify immunoprecipitated proteins after samples were separated using SDS-PAGE.

### Label-free proteomics analysis

For proteomic analysis, proteins were isolated from the cauda epididymis sperm (Genechem). In short, materials were analyzed using a nanoElute (Bruker) system that was connected to a timsTOF Pro (Bruker) mass spectrometer that had a CaptiveSpray source. MaxQuant software 1.6.17.0 was used to analyze the mass spectrometry data. Normalized spectral protein intensity (LFQ intensity) was used to calculate protein abundance. Proteins exhibiting a fold change ≥ 1.2 and a *p*-value < 0.05 (Student’s *t* test) were considered to be the differentially expressed proteins. An online tool for data analysis and visualization, https://www.bioinformatics.com.cn (last viewed on December 10, 2024), was used to plot the heatmap.

### Non-targeted metabolomic sequencing analysis

Sperm samples from six adult WT and six *Lrrc71* KO mice were analyzed, with up to 1 × 10^7^ sperm quantified per sample. Non-targeted sperm metabolomics analysis was performed by Shanghai Genechem Co, Ltd. In short, glass beads were used to homogenize the sperm samples, which were then extracted using liquid nitrogen freeze-thaw cycles and acetonitrile/methanol/water (2:2:1, v/v/v). Following centrifugation and drying, the extracts were filtered through a 0.22 μm membrane after being reconstituted in 0.1% formic acid-acetonitrile containing 4 ppm 2-chlorophenylalanine (internal standard). An ACQUITY UPLC HSS T3 column (2.1 × 100 mm, 1.8 μm) was used for chromatographic separation on a Vanquish UHPLC system at 40 °C with a flow rate of 0.3 ml/min. The mobile phases were 5 mM ammonium formate/acetonitrile (negative mode) and 0.1% formic acid in water/acetonitrile (positive mode) with a gradient elution (10–98% organic phase over 8 min). An Orbitrap Exploris 120 was used for MS analysis in Full MS-ddMS^2^ mode (MS^1^ resolution: 60,000; MS^2^ resolution: 15,000). ProteoWizard was used to convert raw data into mzXML format. XCMS was then used to analyze the data for feature detection, alignment, retention time correction, and area normalization. Metabolites were identified by matching accurate mass and MS/MS spectra against HMDB, MassBank, KEGG, and LipidMaps databases. Multivariate statistical analyses (PCA, PLS-DA, OPLS-DA) were performed to screen differential metabolites (*p* < 0.05, VIP > 1). Pathway enrichment analysis was conducted using MetaboAnalyst, and results were visualized with KEGG Mapper.

### Whole-exome sequencing and Sanger sequencing

The First Affiliated Hospital of Anhui Medical University provided 725 Chinese males (20–45 years old) with asthenoteratozoospermia and 400 men who were normally fertile for the WES study. Peripheral blood genomic DNA was isolated using the QIAGEN DNeasy Kit #69504 and then submitted to WES. The patient and his parents underwent Sanger sequencing with the primers forward 5′-TGTCACGGGTGAGGTAGCACTG-3′ and reverse 5′-CTCTGCTCCATAGAGTCGGGGT-3′ to confirm the discovered LRRC71 mutation (c.1358A >G, p.N453S).

### Statistical analysis

SPSS 25.0 was used to analyze all statistical data. The results are shown as mean ± SD, and each experiment was conducted at least three times. The Mann-Whitney U test was used to compare the two groups when the data did not follow a normal distribution, whereas Student's *t* test was used when it did. When the *p*-values were less than 0.05 (∗), 0.01 (∗∗), and 0.001 (∗∗∗), the results were deemed significant.

## Data availability

The data is available from the corresponding author upon reasonable request. The mass spectrometry proteomics data have been deposited to the ProteomeXchange Consortium (https://proteomecentral.proteomexchange.org) *via* the iProX partner repository with the dataset identifier PXD077837.

## Supporting information

This article contains supporting information.

## Conflict of interest

The authors declare that they have no conflicts of interest with the contents of this article.
