# Supplementary material for: LRRC71 is essential for sperm motility, fertilization, and male fertility
Source: J Biol Chem. 2026 May 20;302(7):113176. doi: 10.1016/j.jbc.2026.113176 (PMC13279179; doi:10.1016/j.jbc.2026.113176)
Supplement: Table S1 [file mmc1.docx]

**Table S1. Information about the antibodies used in this study.**

| Antibodies | Source | Identifier(reference) |
| --- | --- | --- |
| Rabbit polyclonal anti-LRRC71 antibody | This study | N/A |
| Rabbit monoclonal anti-PCNA antibody | Proteintech | 10205-2-AP |
| Rabbit polyclonal anti-histone gamma-H2AX antibody | Affinity | AF3187 |
| Rabbit polyclonal anti-SOX9 antibody | Sigma | ZRB5535 |
| Rabbit monoclonal anti-Tmprss12 antibody | Abclonal | A9126 |
| Rabbit polyclonal anti-GK2 antibody | Abclonal | A17488 |
| Rabbit polyclonal anti-HK1 antibody | Abclonal | A0546 |
| Mouse polyclonal anti-ADAM3 antibody | SANTA CRUZ | Sc-365288 |
| Rabbit polyclonal anti-SPACA1 antibody | Proteintech | 12829-1-AP |
| Rabbit polyclonal anti-ACSS1 antibody | Abclonal | A15007 |
| Rabbit polyclonal anti-LDHC antibody | Abclonal | A15003 |
| Rabbit polyclonal anti-UQCRFS1 antibody | Abclonal | A14609 |
| Rabbit polyclonal anti-ATPB antibody | Abclonal | A5769 |
| Rabbit polyclonal anti-MTCO1 antibody | Abclonal | A17889 |
| Rabbit polyclonal anti-SDHB antibody | Abclonal | A10821 |
| Rabbit polyclonal anti-NDUFS2 antibody | Abclonal | A12858 |
| Mouse monoclonal anti-Alpha Tubulin antibody | Proteintech | 66031-1-lg |
| Rabbit polyclonal anti-AK7 antibody | Solarbio | K117223p |
| Rabbit polyclonal anti-AK8 antibody | Absin | Abs151616 |
| Rabbit polyclonal anti-SEPTIN4 antibody | Proteintech | 12476-1-AP |
| Mouse monoclonal anti MYC-tag antibody | Proteintech | 60003-2-Ig |
| Rabbit polyclonal anti HA-tag antibody | Proteintech | 51064-2-AP |
