# Supplementary material for: LRRC71 is essential for sperm motility, fertilization, and male fertility
Source: J Biol Chem. 2026 May 20;302(7):113176. doi: 10.1016/j.jbc.2026.113176 (PMC13279179; doi:10.1016/j.jbc.2026.113176)
Supplement: Table S2 [file mmc2.docx]

**Table S2. Primers used in this study.**

| Primer sequences for genotyping | |
| --- | --- |
| Lrrc71_Fw#1 | TCCACGAGTCTCCTTCTAAGTGTTAAG |
| Lrrc71_Rv#1 | TGCTAAGACCAAAGCTGAAAATGC |
| Lrrc71_Rv#2 | CGCACACGTTTTACAAGAGAGTTTC |
| Primer sequences for RT-PCR |  |
| Lrrc71_Fw | CACTTTCATCGCCCTTTTGC |
| Lrrc71_Rv | GACAAGTGAACAATCGTGCTG |
| Gapdh_Fw | GTCAAGGCCGAGAATGGGAA |
| Gapdh_Rv | CTCGTGGTTCACACCCATCA |
