# Supplementary material for: LRRC71 is essential for sperm motility, fertilization, and male fertility
Source: J Biol Chem. 2026 May 20;302(7):113176. doi: 10.1016/j.jbc.2026.113176 (PMC13279179; doi:10.1016/j.jbc.2026.113176)
Supplement: Table S3 [file mmc3.docx]

**Table S3. Outcomes of ICSI treatment in mice**

| **Mouse** | **Oocytes injected** | **Fertilization rate (%)** | **2-cell (%)** | **Blastocysts (%)** | **Implanted embryos** | **Offspring number** |
| --- | --- | --- | --- | --- | --- | --- |
| WT#1 | 26 | 100 (26/26) | 100 (26/26) | 92.3 (24/26) | 24 | 3 |
| WT#2 | 26 | 100 (26/26) | 100 (26/26) | 88.5 (23/26) | 23 | 2 |
| WT#3 | 28 | 100 (28/28) | 96.4 (27/28) | 77.8 (21/27) | 21 | 4 |
| KO#1 | 17 | 100 (17/17) | 88.2 (15/17) | 100 (15/15) | 15 | 0 |
| KO#2 | 16 | 100 (16/16) | 100 (16/16) | 87.5 (14/16) | 14 | 3 |
| KO#3 | 19 | 100 (19/19) | 94.7 (18/19) | 88.9 (16/18) | 16 | 0 |
