# Supplementary material for: LRRC71 is essential for sperm motility, fertilization, and male fertility
Source: J Biol Chem. 2026 May 20;302(7):113176. doi: 10.1016/j.jbc.2026.113176 (PMC13279179; doi:10.1016/j.jbc.2026.113176)
Supplement: Supplementary Figures [file mmc4.docx]

**Supplementary Figures and figure legends**

**Figure S1. Expression of LRRC71 in human and mouse tissues.**

**
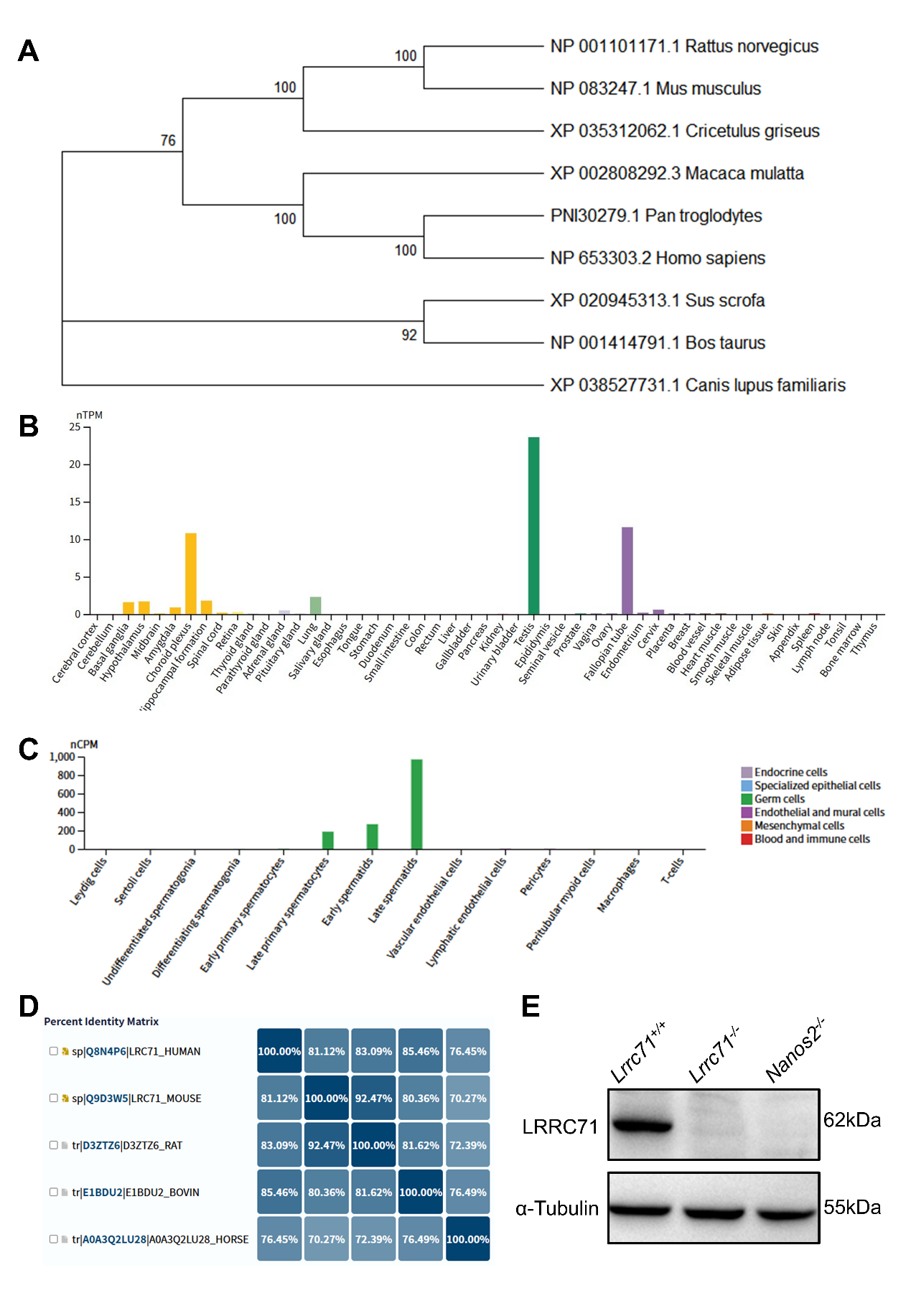
**

**(A)** Phylogenetic tree illustrating the evolutionary divergence of LRRC71 across different animal species. **(B)** The mRNA expression levels of *LRRC71* across multiple human tissues. Data were obtained from the Human Protein Atlas (RNA-seq). Y-axis indicates nTPM (normalized protein-coding transcripts per million). **(C)** *LRRC71*-enriched cell types in the human testis. Data were derived from the Human Protein Atlas (single-cell RNA sequencing). Y-axis represents nCPM (normalized counts per million). (D) Percent identity matrix of 5 mammalian LRRC71 orthologs. **(E)** LRRC71 protein expression in testicular tissue from *Nanos2* knockout mice.

**Figure S2. Assessment of LRRC71 localization in HeLa, NIH3T3, GC1-spg and GC2-spd cells.**

**
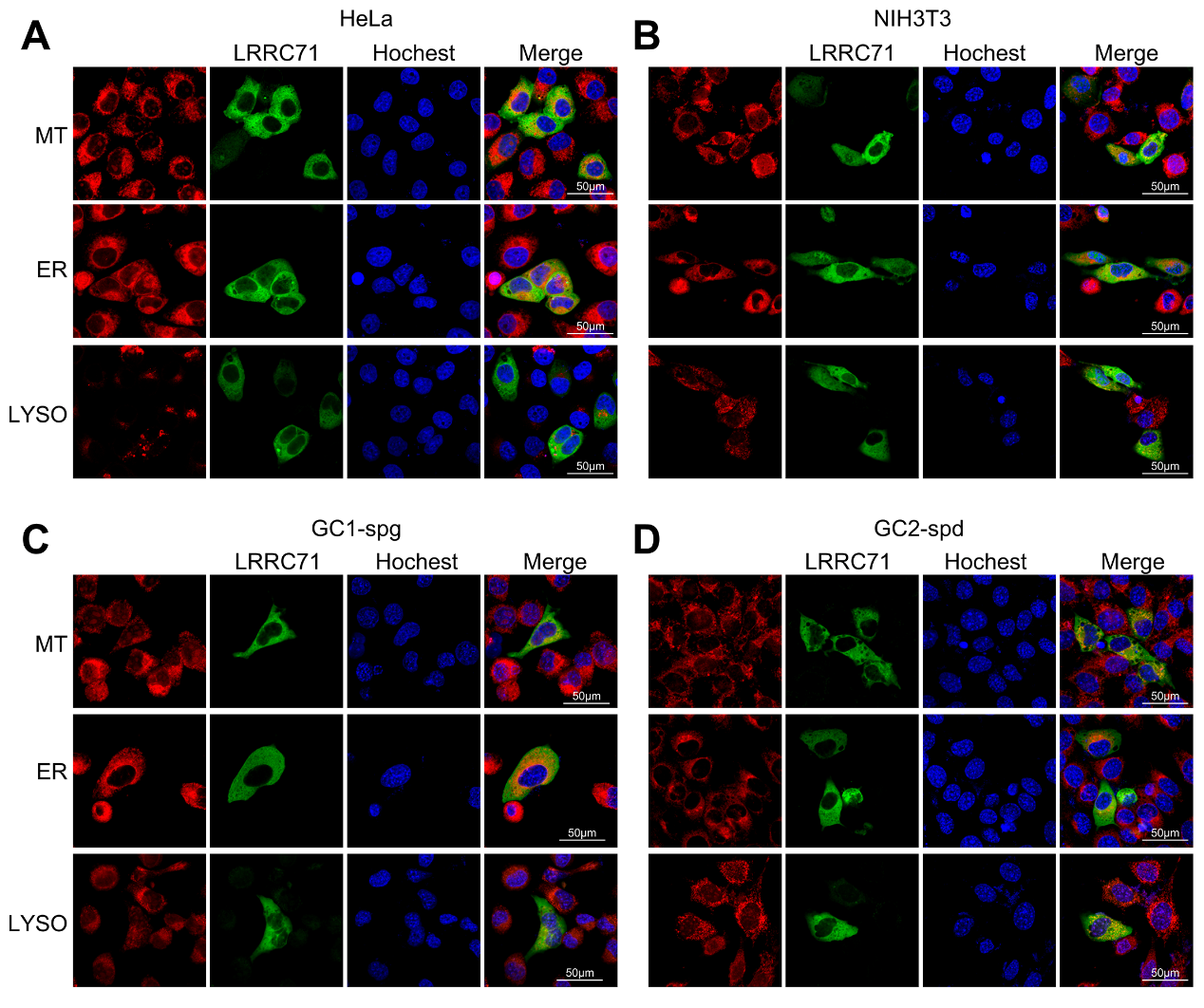
**

**(A-D)** Co-localization analysis was conducted using LRRC71-EGFP-expressing **HeLa, NIH3T3, GC1-spg and GC2-spd cells** stained with organelle-specific markers for endoplasmic reticulum (ER), mitochondria (MT) and lysosomes (LYSO). Scale bar is 50 μm.

**Figure S3. Histological analysis of oviduct and testes.**

**
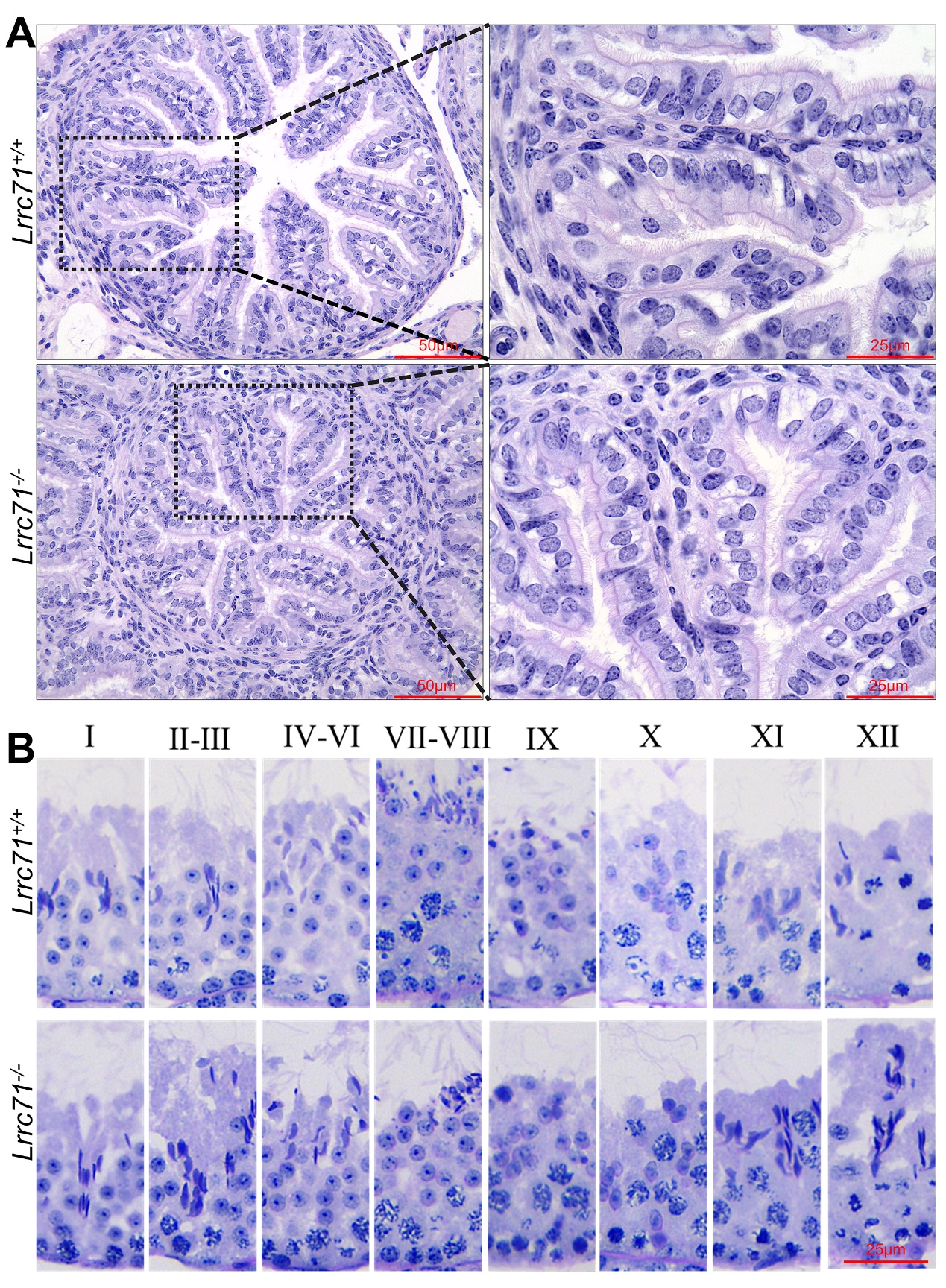
**

**(A)** HE staining of *Lrrc71^+/+^* and *Lrrc71^−/−^* oviducts. Scale bar is 50 μm (left panel), and 25 μm (right panel). **(B)** Different epithelium stages of seminiferous tubules in PAS-stained *Lrrc71^+/+^* and *Lrrc71^−/−^* testes. Scale bar is 25 μm.

**Figure S4. Assessment of germ cells and Sertoli cells in the testes of *Lrrc71^−/−^* mice.**

**
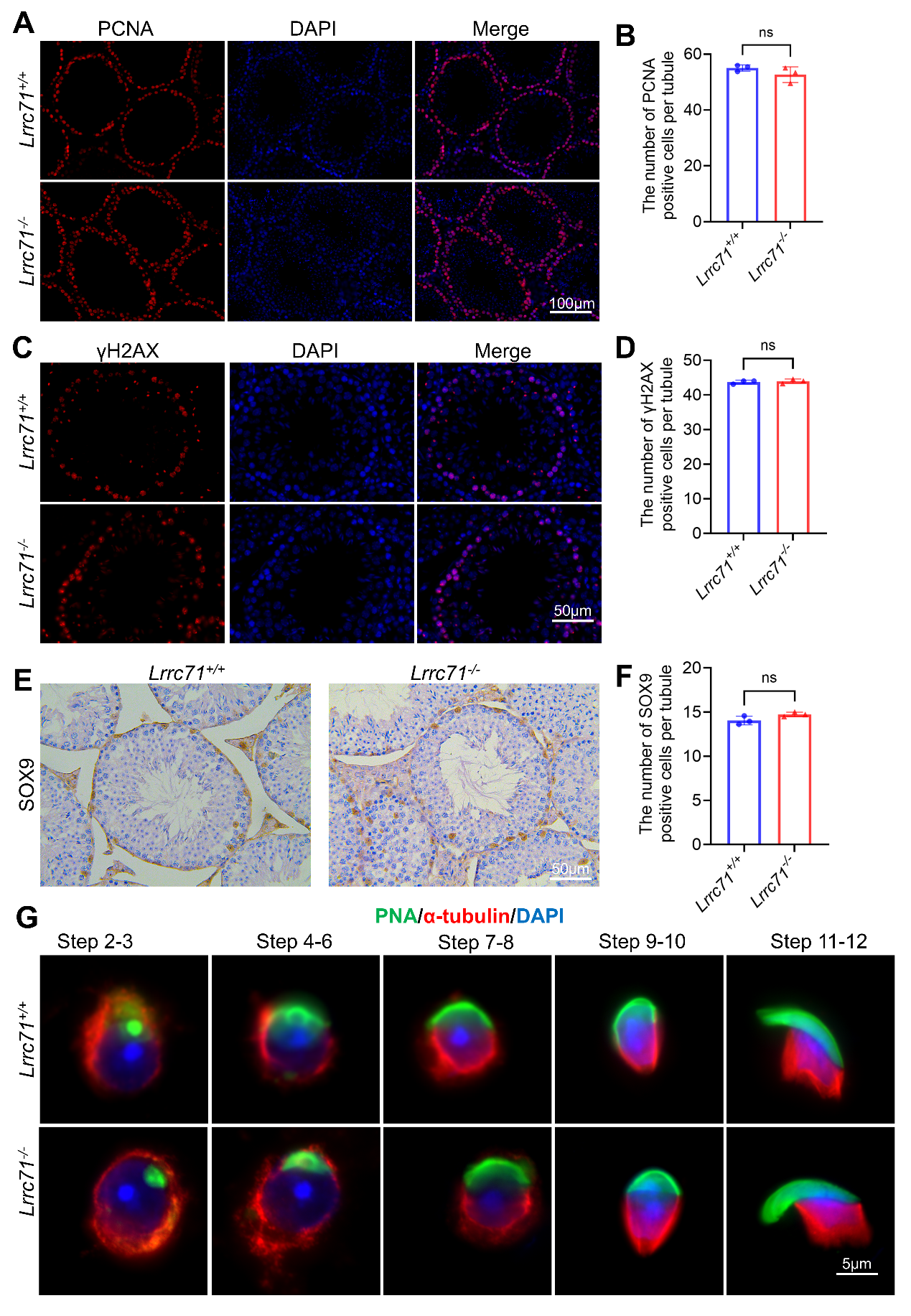
**

**(A-B)** Immunofluorescence (IF) analysis of PCNA-positive cells in the testes of *Lrrc71^+/+^* and *Lrrc71^−/−^* mice (n=3). Scale bar is 100 μm. Student’s *t*-test. ns: no significant difference. **(C-D)** IF analysis of γH2AX-positive cells in the testes of *Lrrc71^+/+^* and *Lrrc71^−/−^* mice (n=3). Scale bar is 50 μm. Student’s *t*-test. ns: no significant difference. **(E-F)** IF analysis of SOX9-positive cells in the testes of *Lrrc71^+/+^* and *Lrrc71^−/−^* mice (n=3). Student’s *t*-test. ns: no significant difference. Scale bar is 50 μm. **(G)** IF analyses of PNA and α-tubulin in the testicular suspension of *Lrrc71^+/+^* and *Lrrc71^−/−^* mice. Scale bar is 5 μm. All data are presented as mean ± SD.
